# Proximity Labeling Facilitates Defining the Proteome Neighborhood of Photosystem II Oxygen Evolution Complex in a Model Cyanobacterium

**DOI:** 10.1016/j.mcpro.2022.100440

**Published:** 2022-11-08

**Authors:** Zhen Xiao, Chengcheng Huang, Haitao Ge, Yan Wang, Xiaoxiao Duan, Gaojie Wang, Limin Zheng, Jinghui Dong, Xiahe Huang, Yuanya Zhang, Hongyu An, Wu Xu, Yingchun Wang

**Affiliations:** 1 State Key Laboratory of Molecular Developmental Biology, Innovation Academy for Seed Design, CAS, Institute of Genetics and Developmental Biology, Chinese Academy of Sciences, Beijing, China; 2 College of Advanced Agricultural Sciences, University of Chinese Academy of Sciences, Beijing, China; 3 Department of Chemistry, University of Louisiana at Lafayette, Lafayette, Louisiana, USA

**Keywords:** APEX2, proximity labeling, proteomics, cyanobacteria, Synechocystis, *Synechocystis*, *Synechocystis* sp. PCC6803, WT, Wildtype, APEX, Ascorbate peroxidase, BP, Biotin phenol, H_2_O_2_, Hydrogen peroxide, OEC, oxygen evolution complex, PS I, Photosystem I, PS II, Photosystem II, IMP, Integral membrane protein, WCL, Whole cell lysate, TM, Transmembrane domain, Cyt *b*_*6*_/*f*, cytochrome *b*_*6*_*/f*, Sec, Secretory, Tat, Twin-arginine translocation

## Abstract

Ascorbate peroxidase (APEX)-based proximity labeling coupled with mass spectrometry has a great potential for spatiotemporal identification of proteins proximal to a protein complex of interest. Using this approach is feasible to define the proteome neighborhood of important protein complexes in a popular photosynthetic model cyanobacterium *Synechocystis* sp. PCC6803 (hereafter named as *Synechocystis*). To this end, we developed a robust workflow for APEX2-based proximity labeling in *Synechocystis* and used the workflow to identify proteins proximal to the photosystem II (PS II) oxygen evolution complex (OEC) through fusion APEX2 with a luminal OEC subunit, PsbO. In total, 38 integral membrane proteins (IMPs) and 93 luminal proteins were identified as proximal to the OEC. A significant portion of these proteins are involved in PS II assembly, maturation, and repair, while the majority of the rest were not previously implicated with PS II. The IMPs include subunits of PS II and cytochrome *b*_*6*_/*f*, but not of photosystem I (except for PsaL) and ATP synthases, suggesting that the latter two complexes are spatially separated from the OEC with a distance longer than the APEX2 labeling radius. Besides, the topologies of six IMPs were successfully predicted because their lumen-facing regions exclusively contain potential APEX2 labeling sites. The luminal proteins include 66 proteins with a predicted signal peptide and 57 proteins localized also in periplasm, providing important targets to study the regulation and selectivity of protein translocation. Together, we not only developed a robust workflow for the application of APEX2-based proximity labeling in *Synechocystis* and showcased the feasibility to define the neighborhood proteome of an important protein complex with a short radius but also discovered a set of the proteins that potentially interact with and regulate PS II structure and function.

## Highlights

• A robust proximity labeling workflow was developed for *Synechocystis*.
• Identification of proteins proximal to the PSII subunit PsbO in *Synechocystis*.
• 38 integral membrane proteins and 93 luminal proteins are proximal to PsbO.

## Graphical Abstract

**Figure:**
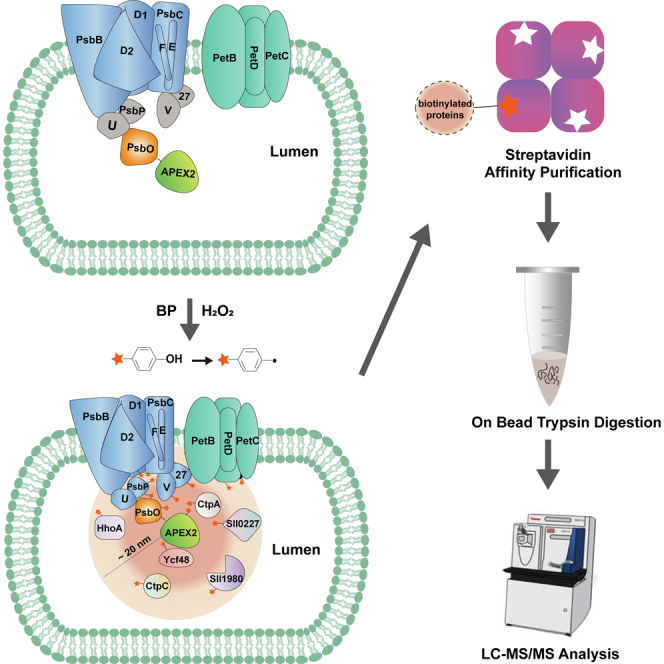

## In Brief

An effective workflow for APEX2-based proximity labeling in the model cyanobacterium *Synechocystis* sp. PCC 6803 was developed. Proximity labeling of proteins proximal to the photosystem II oxygen evolution complex by PsbO-APEX2 resulted in proteomic identification of 38 integral thylakoid membrane proteins and 93 luminal proteins, thereby defining the proteome neighborhood of photosystem II in the luminal side.

Cyanobacteria are a group of gram-negative bacteria capable of oxygenic photosynthesis. Cyanobacteria thrive in almost all kinds of terrestrial and aquatic habitats on the earth and are an important component of our ecosystem by providing a significant portion of oxygen in the atmosphere (1). Cyanobacteria highly resembles the chloroplasts of the higher plants in both structure and function, and it is believed that the latter are derived from the former through endosymbiosis (2), and therefore, cyanobacteria are frequently used as model organisms for studying photosynthesis. Cyanobacteria have recently caught more attention because of their capability of photoautotrophic growth and the potential to serve as cell factory to produce clean and renewable biofuels and high-value compounds (3, 4, 5).

To better use cyanobacteria both as model organisms for photosynthesis research and as green *E.coli* for industrial production, it is important to understand how diverse types of biological and physiological processes and metabolic pathways are operated and coordinated in a single compartment. This is evident as photosynthesis must be coordinated with carbon and nitrogen metabolisms, and production of renewable biofuels and high-value compounds need metabolic engineering that diverts metabolic flux to the target metabolites. Proteins are essential players in these processes and pathways, and they locate and operate in a single or multiple subcellular compartments. Determining the cellular locations of proteins is critically important to the understanding how the processes and pathways they are involved are regulated and coordinated in time and space. Therefore, mounting efforts have been invested on the determination of protein locations in cyanobacteria, particularly at a proteome level (6, 7, 8, 9, 10, 11, 12). To this end, the major subcellular compartments of cyanobacteria such as thylakoid membrane, plasma membrane, outer membrane, periplasmic space, and even the extracellular proteome have been biochemically isolated, and the protein components in each compartments have been identified using either 2D-MS or LC-MS approaches (6, 7, 8, 9, 10, 11, 12). Using these approaches, the majority of proteins encoded by the genome of a model cyanobacterium *Synechocystis* have been determined for their subcellular locations, and a proteome atlas has been constructed based on such information (11). Though the atlas provides subcellular localization information for the majority of proteins, the spatial resolution of the atlas is still insufficient for defining protein residents in a specific domain of a subcellular compartment. It is documented that thylakoid membrane and plasma membrane as well are not homogenous and contain different domains with different protein and lipid compositions (13, 14, 15). It is conceivable that the major macromolecular protein complexes such as photosystem I (PS I) and photosystem II (PS II) may reside at particular domains with specific proteins in the neighborhood and thereby to maintain their structural stability and optimal function. Moreover, the atlas does not contain information for the proteins in thylakoid lumen, a subcellular compartment of cyanobacteria that has never been isolated for proteome analysis due to the lack of an effective approach for biochemical separation and purification of the compartment.

Recent technological innovations in proximity labeling technologies allow labeling and capturing proteins in the nearby of target proteins (16). Among them, peroxidase proximity labeling with ascorbate peroxidase (APEX) and its upgraded version APEX2 are of particular interest because their short reaction time and small labeling radius (16, 17), which are important for spatiotemporally defining the neighborhood proteome of the protein of interest. It is possible to genetically fuse APEX2 with a subunit of the major macromolecular protein complexes on the thylakoid membrane such as PS I and PS II or a protein in thylakoid lumen and to identify proteins in the neighborhood of the corresponding protein complexes or in the thylakoid lumen. To date, APEX2 has been successfully used in different systems including yeast, animal cell lines, and tissues (18, 19, 20). In cyanobacteria, only one study was available regarding APEX2 application. In that report, APEX2 was targeted to the thylakoid lumen of *Synechococcus* sp. PCC 7002 through fusion with a PS II subunit PsbU, and 123 biotinylated proteins were subsequently identified as luminal proteins (21).

Herein, we firstly optimized a workflow that can serve as a general protocol for APEX2 application in cyanobacterium *Synechocystis*, a popular model organism for photosynthesis research because of its completely sequenced genome and the natural ability of transformation (22, 23). Then we used the protocol to identify proteins proximal to the extrinsic oxygen evolution complex (OEC) of PS II through fusion of APEX2 with PsbO, an OEC subunit. A number of integral thylakoid membrane proteins and luminal proteins were identified. A small portion of the identified proteins are known to be proximal to PS II or in lumen, whereas the other majority are not previously known representing novel targets potentially involved in regulation of PS II structure and function.

## Experimental Procedures

### Antibodies

The primary antibodies for glyceraldaldehyde-3-phosphate dehydrogenase 2, CpcB were provided by PhytoAB. The antibody for HA was purchased from MBL. The antibody for D1 was purchased from Agrisera. The antibody for GFP was provided by Abcam. The antibody for biotin was provided by Cell Signaling. The streptavidin-HRP was provided by Thermo Scientific.

### Cell Culture

*Synechocystis* cells (the glucose-tolerant strain) were grown in liquid BG11 medium on a shaker (200 rpm) at 30°C with a photosynthetic photon flux density of 50 μmol m^−2^ s^−1^. The concentration of cells in liquid culture was estimated from the absorbance at 730 nm (*A*_730_) using a SmartSpec Plus spectrophotometer (Bio-Rad).

### Mutant Generation

The knock-in strains of *Synechocystis* expressing APEX2/HA-tagged target proteins were generated as described previously (24). Briefly, a ∼1 kb DNA fragment of target genes spanning from about 500 bp to the 5′ prime of the stop codon to about 500 bp to the 3′ prime of the stop codon were cloned by PCR using the primers listed in supplemental Table S1. A DNA fragment containing APEX2 or HA tag and a kanamycin-resistance gene cassette was inserted into the target gene fragment before the stop codon. The final constructs were used to transform wildtype (WT) *Synechocystis*, and the knock-in strains were selected on solid BG11 plates containing kanamycin. The fully segregated strains were confirmed by PCR as previously described (25).

### Biotin-Phenol Labeling

The cyanobacterial culture at the exponential phase (*A*_730_ = 1.0) was diluted to a cell density of *A*_730_ = 0.2 and grown until *A*_730_ = 0.8. Cells were harvested by centrifugation at 4000*g* for 5 min and resuspended in BG11 containing 2 mM biotin phenol (BP) and incubated at 30 °C for 10 min. H_2_O_2_ was then added to a final concentration of 2 mM, and the labeling reaction was allowed to continue for 1 min. The reaction was quenched by replacing the medium with ice cold quenching buffer (TBS supplemented with 1 mM CaCl_2_, 10 mM sodium ascorbate, 1 mM sodium azide, and 1 mM Trolox).

### Enrichment of Biotin-Labeled Proteins With Streptavidin

Cells were lysed in a RM buffer (50 mM Tris, 150 mM NaCl, 1% NP40, 0.1% deoxycholate, 1 mM EDTA, pH 7.5, and 0.5 mM phenylmethanesulfonyl fluoride (Sigma-Aldrich) with a bead beater, and the insoluble debris was removed by centrifugation with 5000g for 30 min at 4 °C. After precipitation with methanol and chloroform (26), total proteins were washed with methanol to remove pigments, lipids, and residual chloroform. The precipitated proteins were air-dried before re-solubilization with 0.1% sodium dodecyl sulfate (SDS) in 0.1 M Tris-HCl (pH 7.6). The protein concentrations were measured using a BCA protein assay kit (Thermo Scientific). To enrich biotinylated proteins, 4 mg of proteins were added to 100 μl of streptavidin-agarose beads (Thermo Scientific), which were then rotated and incubated overnight at 4 °C. The beads were subsequently washed once with RM buffer, twice with PBST, once with 1 M KCl. For streptavidin blotting analysis, biotinylated proteins were then eluted by boiling the beads in 50 μl of 3× protein loading buffer supplemented with 20 mM DTT and 2 mM biotin and run on SDS-PAGE gel.

### On-Bead Trypsin Digestion and Desalting

On-bead trypsin digestion was performed in the same way as described previously (27). Briefly, proteins bound to streptavidin beads were first reduced by adding DTT to a final concentration of 10 mM and incubating at 37 °C for 1h with shaking and then alkylated by adding iodoacetamide (Sigma-Aldrich) to a final concentration of 55 mM and incubating at 25 °C for 45 min with shaking. Finally, trypsin (Sigma-Aldrich) was added to digest proteins by overnight incubation at 37 °C with shaking. The peptides were acidified by acetic acid and desalted using C18 StageTips (28). Desalted peptides were dried with a Speed-Vac concentrator, stored at −20 °C, and resuspended in 0.1% formic acid (FA) immediately before LC-MS/MS.

### Mass Spectrometry

For MS analysis, the peptides were analyzed by an LTQ Elite mass spectrometer (Thermo Fisher Scientific) coupled online to an Easy-nLC 1000 in a data-dependent mode. Briefly, peptide sample was injected into a 25 cm length, 75 μm inner diameter capillary analytic column packed with C18 particles of 5 μm diameter (SunChrom). The LC gradient was composed of 3% to 8% buffer B (buffer B contained 100% ACN and 0.1% FA, whereas buffer A contained 0.1% FA) for 10 min, 8% to 20% buffer B for 60 min, 20% to 30% buffer B for 8 min, 30% to 100% buffer B for 2 min, and 100% buffer B for 10 min. The flow rate was set at 600 nl/min. The source voltage was set at 2.5 KV, and the current was set at 100 μA. MS measurement was performed in positive ion mode. The precursors were measured by survey scans in the Orbitrap with a mass range of 300 to 1800 m/z. The 20 most abundant precursor ions from each survey scan were isolated and fragmented by collisional dissociation for MS/MS analysis. The duration of dynamic exclusion was set to 30 s to prevent repeat identification of peptide ions within the time duration.

### Database Search

The raw MS files were searched against the *Synechocystis* proteome sequence database appended with 248 common contaminations using the software MaxQuant (version 1.6.0.16) (29). The database containing 3672 entries was downloaded from CyanoBase (ftp://ftp.kazusa.or.jp/pub/CyanoBase/Synechocystis, released on 5/11/2009). Trypsin/P was set as the protease for protein digestion, and up to two missed cleavages were allowed. N-terminal acetylation and methionine oxidation were chosen as the variable modifications and cysteine carbamidomethylation was chosen as the fixed modification. For precursor ions, the mass tolerance was specified at 20 ppm for the first search and 4.5 ppm for the main search. For fragment ions, the mass tolerance was set to 20 ppm. The 1% false discovery rate was set at both the peptide and protein levels. The minimum scores for unmodified peptides and modified peptides were set to 15 and 40, respectively. The match between runs was enabled. All other parameters were set to default values of MaxQuant.

### Experimental Design and Statistical Rationale

Four label-free quantitative experiments were performed to identify of proteins proximal to PsbO. The mutant cells expressing PsbO-APEX2 incubated with BP and H_2_O_2_ were used as the experimental group. The mutant cells expressing PsbO-APEX2 incubated with H_2_O_2_ but without BP, the WT, and the mutant cells expressing Zwf-APEX2 incubated with BP and H_2_O_2_ were included as the three negative controls. Three biological replicates of each treatment were included. After searching the database, the label-free quantitative intensities were used to calculate the ratios for each protein between the experimental group and the controls. Only proteins identified with MS/MS count >1 in the experimental group were included for all quantitative analyses.

### Bioinformatics and Statistics

Bioinformatic and statistical analyses were mainly performed using the software Perseus, version 1.5.2.6 (30). Enrichment analysis was performed with Fisher’s exact test. A *p*-value<0.05 was used as the cut-off for all statistical analyses.

## Results

### Exogenous Expression of Active APEX2 in Synechocystis

**Fig. 1:**
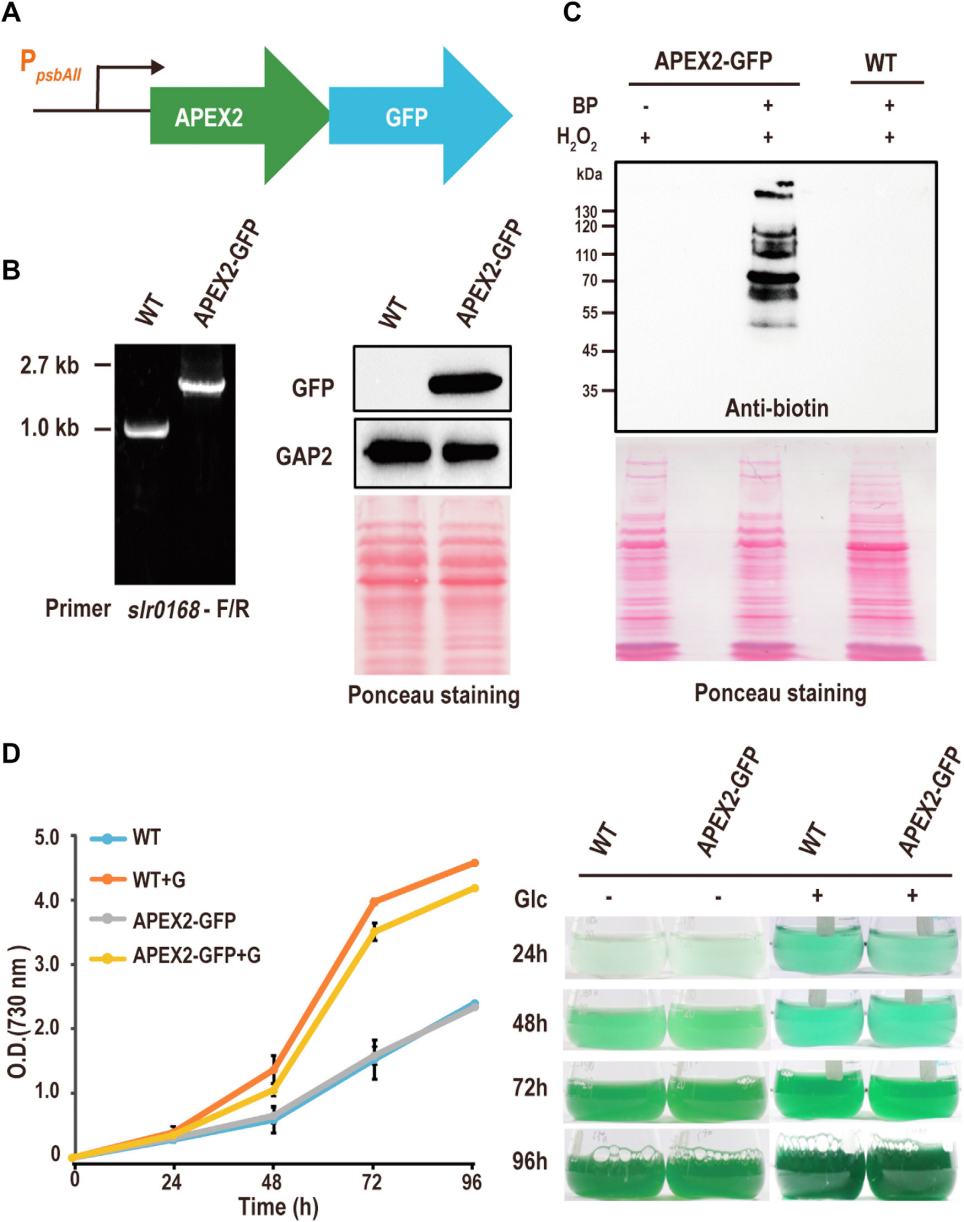
**Expression of active APEX2 in *Synechocystis*.***A*, the diagram depicts the plasmid construct used to express APEX2-GFP fusion protein at a neutral genomic site (*slr0168* locus) under the control of the *psbAII* promoter in *Synechocystis*. *B*, confirmation of the complete segregation of the mutant by PCR (*left panel*) and the expression of APEX2-GFP by Western blotting (*right panel*). The same pair of primers were used to amplify DNA fragments from the WT and the mutant. GAP2 blot and Ponceau staining were included as the loading control. *C*, Western blotting detection of endogenous proteins biotin-labeled by APEX2-GFP. Samples were labeled for 1 min, the BP omitted sample and the WT were included as the negative controls. *D*, growth curves of the mutant and the WT strains measured in the presence (photomixotrophic) or absence (photoautotrophic) of 5 mM glucose under medium light intensity (50 μmol m^−2^s^−1^) (*Left panel*). The cultures were photographed at the indicated time points during the time course (*Right panel*). APEX, ascorbate peroxidase; BP, biotin phenol; GAP2, glyceraldaldehyde-3-phosphate dehydrogenase 2.

To test whether APEX2 is active for proximity labeling in *Synechocystis*, a mutant strain expressing APEX2-GFP under the control of the promoter of *psbAII* (P_*PsbAII*_-APEX2), a gene encoding the PS II D1 protein, was generated by inserting the exogenous DNA fragment into the chromosome through homologous recombination at a neutral site where the ORF *slr0168* resides (Fig. 1*A*) (31). The complete segregation of the mutant was confirmed by PCR, and the expression of APEX2-GFP was detected by Western blotting (Fig. 1*B*). To test the peroxidase activity of APEX2, the mutant cells were incubated with or without BP for 10 min and then treated with 1 mM hydrogen peroxide (H_2_O_2_) to activate the catalytic biotinylation reaction (16). The WT treated with both BP and H_2_O_2_ was also included as the control. The whole cell lysates (WCLs) were probed for biotinylated proteins by Western blotting. Strong biotinylation signals were only detected in the lane for P_*PsbAII*_-APEX2 cells treated with BP, but not in the other lanes (Fig. 1*C*). The data indicate that APEX2 can be exogenously expressed and activated and could be used for proximity labeling in *Synechocystis*. APEX2 catalyzes the reduction of H_2_O_2_ to water by oxidizing ascorbic acid, and the reaction is important for regulating the level of toxic oxidative radicals (32). To investigate whether the exogenous expression of APEX2 causes growth defects in *Synechocystis*, the growth phenotype of the mutant cells was examined. The P_*PsbAII*_-APEX2 showed barely observable growth defect under the autotrophic and mixotrophic growth conditions in comparison with the WT (Fig. 1*D*). Together, these results suggest that active APEX2 could be expressed for biotinylation of the endogenous proteins of *Synechocystis* without causing observable growth defect in the host cells.

### Optimization of the Experimental Conditions for APEX2-Catalyzed Protein Biotinylation

**Fig. 2:**
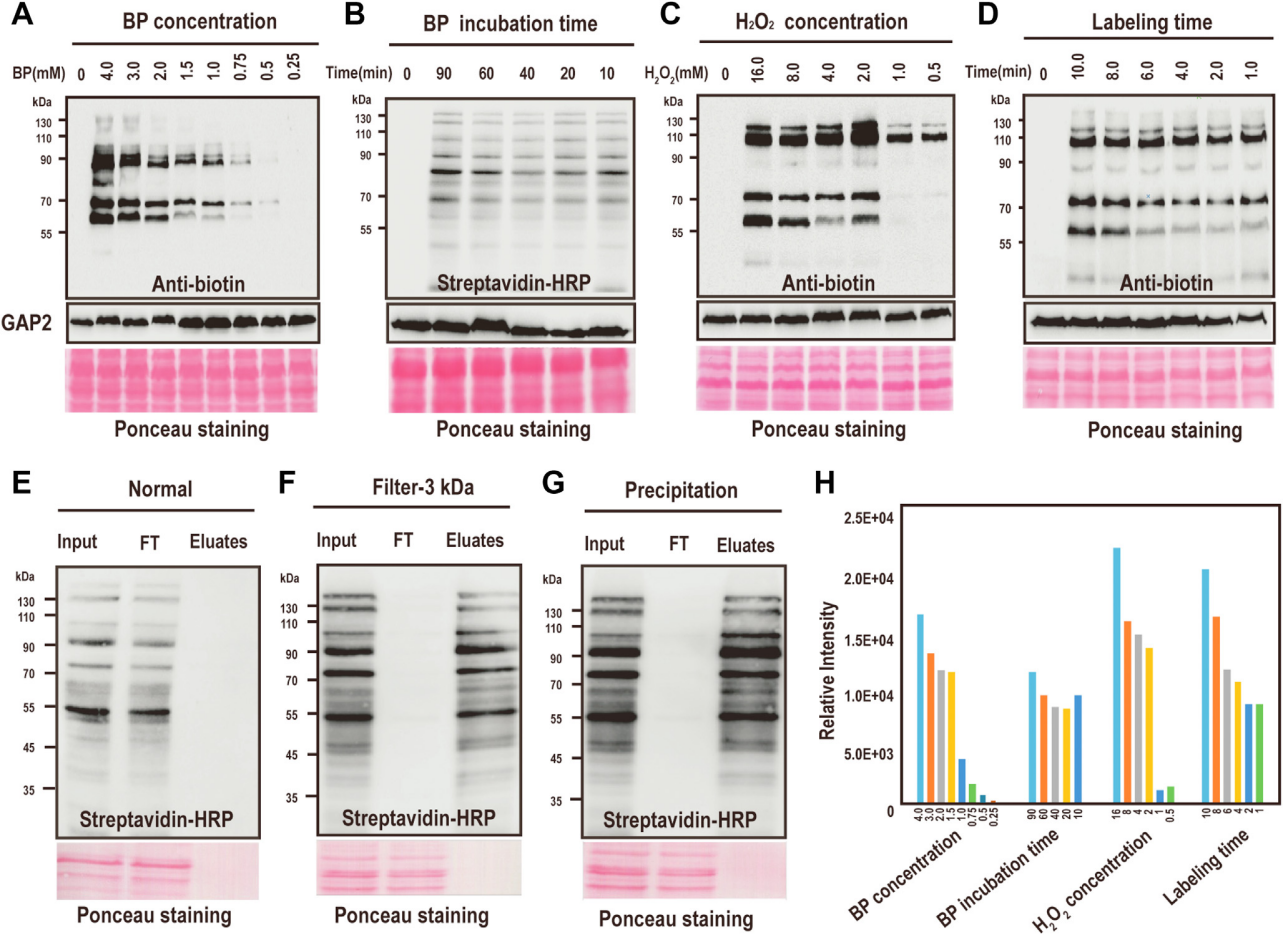
**Optimization of the experimental conditions for APEX2 catalyzed labeling and affinity purification of biotinylated endogenous proteins.***A*–*D*, optimization working conditions for APEX2 including BP concentration (*A*) and incubation time (*B*), H_2_O_2_ concentration (*C*) and labeling time (*D*) at room temperature. Activity and expression of APEX2-GFP fusion proteins were analyzed by streptavidin or anti-biotin Western blotting as indicated. Ponceau staining was used as the loading control. *E*–*G*, optimization of the affinity purification of biotinylated proteins by streptavidin beads. WCLs of P_*PsbAII*_-APEX2 were directly incubated with streptavidin agarose (*E*) or filtered with 3 kDa ultracentrifugal filters and followed by incubation with streptavidin agarose (*F*) or precipitated with methanol/chloroform before incubation with streptavidin agarose (*G*). The input, FT and the eluates were separated by SDS-PAGE and probed for biotinylated proteins by Western blotting. *H*, the bar graph shows the relative intensity of the biotinylated proteins in different conditions shown in (*A*–*D*). Image J was used for the quantitative densitometry analysis on a representative image from each triplicated experiments. APEX, ascorbate peroxidase; FT, flow through; WCLs, whole cell lysates.

To achieve an optimal efficiency of APEX2-catalyzed protein biotinylation in *Synechocystis*, we tested the concentration and incubation time for BP and H_2_O_2_ in the reaction. For mammalian cell culture, 0.5 mM BP was sufficient to label proteins (33). Thus, we tested BP concentrations below or above 0.5 mM, ranging from 0.25 to 4 mM. Western-blotting detection of biotinylated proteins from the WCLs of 10 min BP-treated cells shows that biotinylation is detectable for the cells treated with 0.5 mM BP and increases along with the increasing concentration of the BP. Because BP is expensive and excess BP may affect the subsequent affinity purification as shown below (Fig. 2*E*), we chose the concentration 2 mM instead of a higher one for further experiments (Fig. 2, *A* and *H*). To optimize the time for BP incubation, the cells were incubated with BP from 10 min to 90 min, a time scale that covers the time usually used for mammalian cells (*i.e*., 30 min) (34). Western blotting of the WCLs showed that there was no apparent change of the labeled proteins regardless of the incubation time with BP. Thus, we chose 10 min as the BP incubation time hereafter (Fig. 2, *B* and *H*).

The appropriate H_2_O_2_ concentration is also an important factor for efficient APEX2-catalyzed protein biotinylation. Low H_2_O_2_ may be not sufficient for the reaction, and high H_2_O_2_ may produce free radicals leading to oxidative damage to proteins and membrane lipids. Therefore, we tested H_2_O_2_ concentration ranging from 0.5 to 16 mM to determine the optimal concentration. Weak protein biotinylation can be detected for the cells treated with 0.5 or 1 mM H_2_O_2,_ and strong biotinylation was detected for the cells treated with 2 mM H_2_O_2_. Further increase of H_2_O_2_ concentration did not increase the level of protein biotinylation (Fig. 2, *C* and *H*). Thus, we chose 2 mM as the working concentration for H_2_O_2_. The optimal working time for H_2_O_2_ was also tested because long-time H_2_O_2_ incubation could cause damage to the cells resulting in experimental artifacts. Encouragingly, 1 minute of H_2_O_2_ treatment is sufficient for efficient APEX2-catalyzed biotinylation as detected by the immunoblotting (Fig. 2, *D* and *H*). Together, the experimental condition was optimized for APEX2-GFP–catalyzed protein biotinylation in *Synechocystis*, and the same condition will be used hereafter for the APEX2-based proximity labeling.

### Optimization of the Affinity Purification of Proteins Labeled by APEX2-Catalyzed Biotinylation

Affinity purification of the biotinylated proteins was tried using streptavidin beads. Unexpectedly, the purification was not successful, and no biotin signal was detected in the eluates (Fig. 2*E*). Increasing the amount of the starting material, *i.e*., the WCLs, did not solve the problem. It is suggested that the existence of free BP in the WCLs, which is the leftover of the AEPX2-catalyzed reaction, could competitively inhibit the binding of the biotinylated proteins to the streptavidin beads (35). Existing evidence shows that for removal of free BP from the WCLs, thorough washing of the cells is sufficient for mammalian cell culture or rice protoplasts, whereas an additional filtration step is needed for plant tissue (35). Therefore, we filtered the WCLs using ultrafilters with a 3 kDa molecular weight cutoff before performing streptavidin affinity purification. As expected, the biotinylated proteins in the filtered WCLs successfully bound to streptavidin beads and showed strong signals in the eluates when probed by streptavidin. In contrast, the biotinylation signal was barely displayed in the flow through indicating high efficiency of affinity purification (Fig. 2*F*). Since the preparation of the WCLs and the subsequent affinity purification were performed in a nondenaturing condition, many nonbiotinylated proteins could interact and coprecipitate with the biotinylated proteins. To address this problem, the WCLs were denatured by precipitation with methanol/chloroform and re-solubilized in a buffer containing 0.1% SDS before streptavidin immunoprecipitation. It is notable that the precipitation can also effectively remove the free BP from the sample, making the filtration step avoidable. Successful biotin labeling and purification of biotinylated proteins were confirmed by immunoblotting (Fig. 2*G*). Taking the above in consideration, we chose the precipitation approach to prepare further samples for affinity purification of biotinylated proteins.

### Proximity Labeling of Proteins in the Neighborhood of PsbO-APEX2

**Fig. 3:**
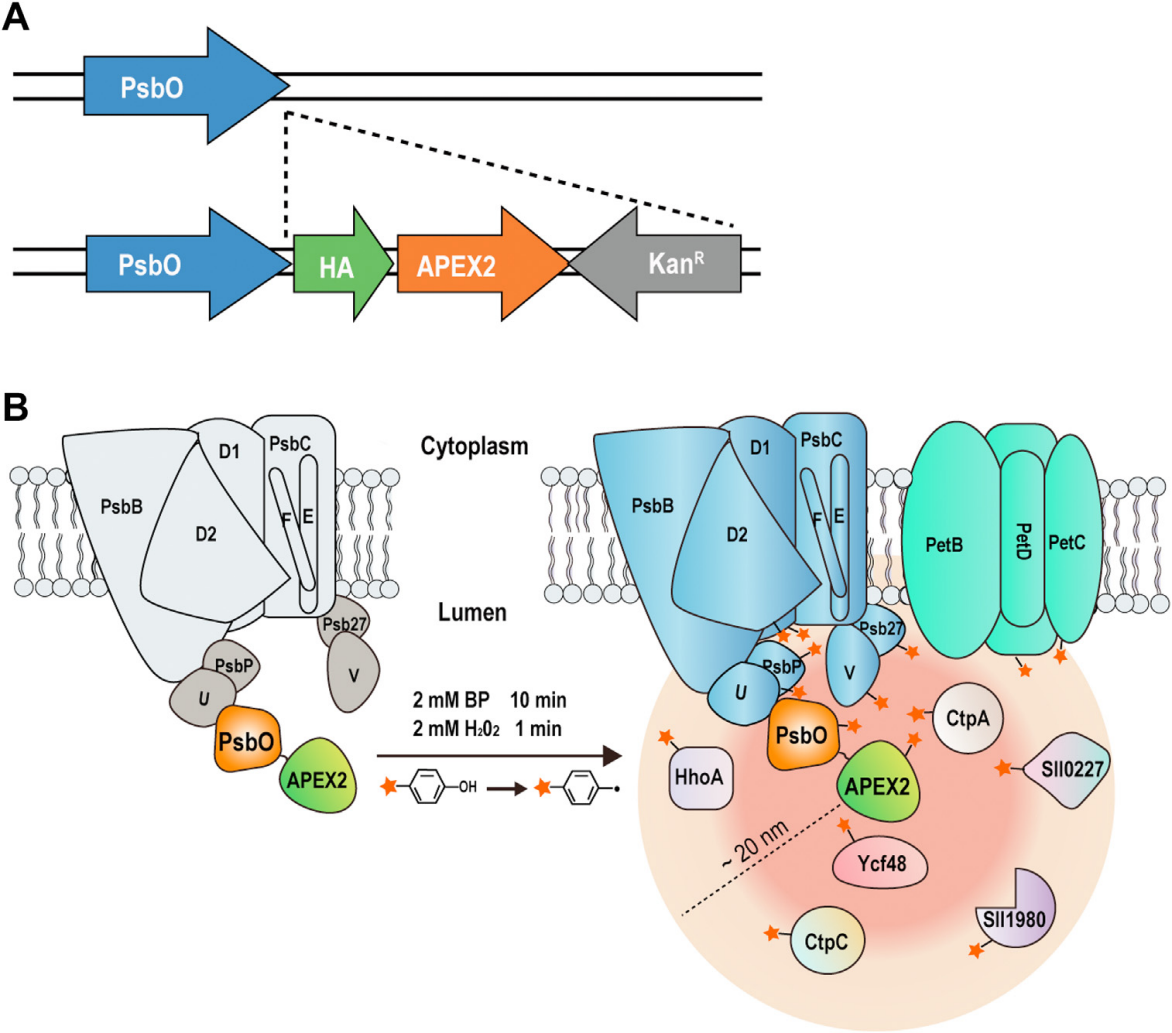
**Proximity labeling of proteins by PsbO-APEX2.***A*, the diagram depicts the plasmid construct used to transform *Synechocystis* for the expression of PsbO-APEX2. *B*, diagram illustration of PsbO-APEX2 catalyzed biotin-labeling of proteins proximal to the PS II OEC complex. Note that both luminal proteins and integral thylakoid membrane proteins with domains exposed to the lumen can be labeled. APEX, ascorbate peroxidase; OEC, oxygen evolution complex; PS II, photosystem II.

**Fig. 4:**
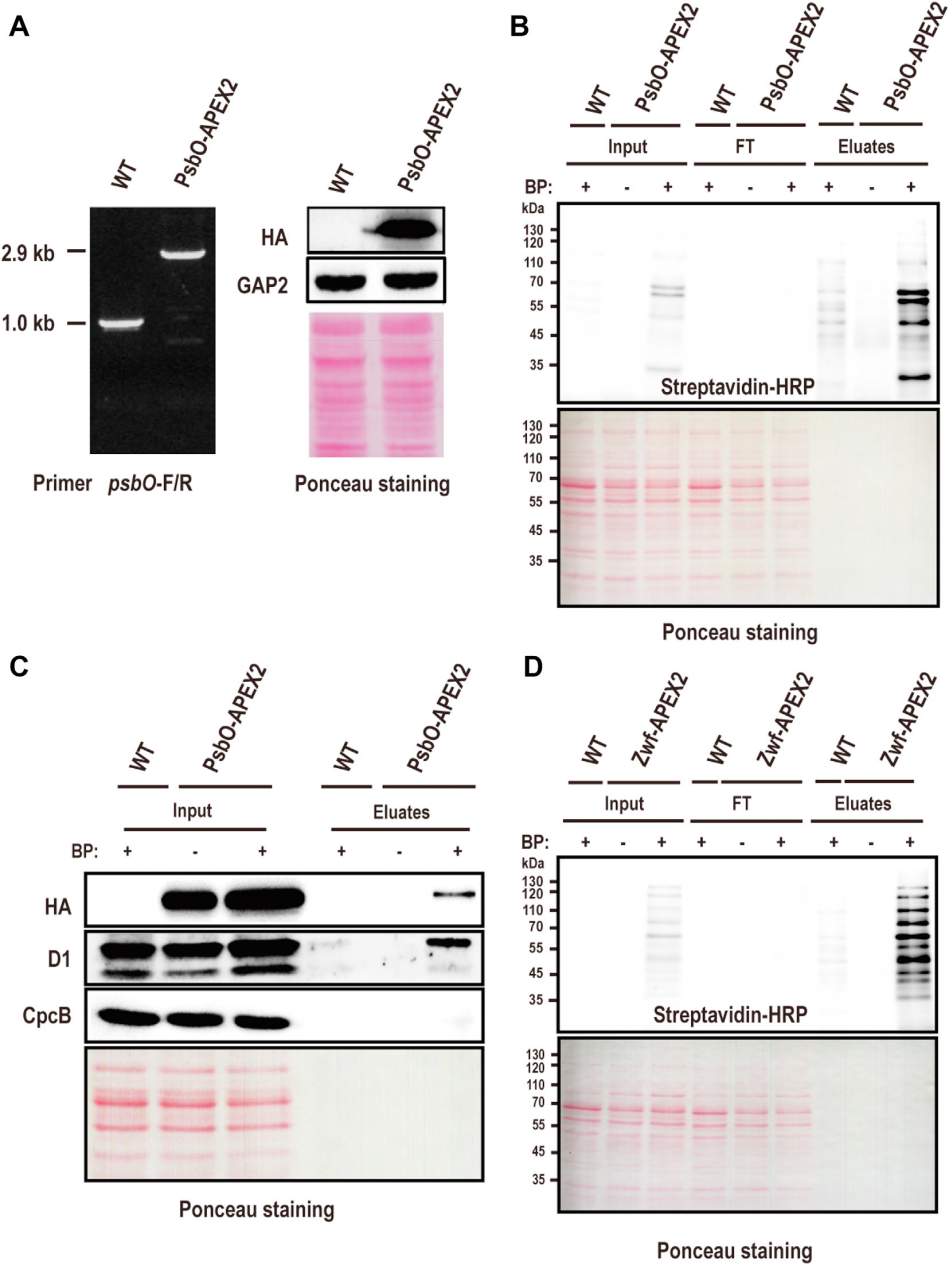
**Affinity purification of proteins labeled by PsbO-APEX2.***A*, the complete segregation and expression of PsbO-APEX2 was confirmed by PCR (*left pane*l) and Western blotting (*right panel*), respectively. Equal loading was shown by Ponceau staining. *B*, affinity purification and Western blotting detection of the biotinylated proteins from the indicated cells incubated with or without BP. The streptavidin-HRP was used detect biotinylated proteins in the input, FT, and the eluates. *C*, Western blotting detection of PsbO-HA-APEX2, D1, and CpcB in both the inputs and the eluates. *D*, same as in (*B*) except that PsbO-APEX2 is replaced by Zwf-APEX2, an additional negative control. APEX, ascorbate peroxidase; BP, biotin phenol; FT, flow through.

To express APEX2 in the thylakoid lumen, a knock-in mutant was generated to express PsbO-APEX2, with an HA tag inserted between the C terminus of PsbO and N terminus of APEX2 (Fig. 3*A*). PsbO is a subunit of the OEC and resides in the thylakoid lumen, and PsbO-APEX2 is expected to label proteins proximal to the PS II OEC complex, which should include luminal proteins and integral thylakoid membrane proteins with domains exposed to lumen (Fig. 3*B*). The complete segregation of the mutant was confirmed by PCR, and the expression of the fusion protein was confirmed by Western blotting using anti-HA antibody (Fig. 4*A*).

PsbO-APEX2-catalyzed proximity labeling and affinity purification of the biotinylated proteins were performed using the optimized condition as described above (Fig. 2). The biotinylated proteins in the WCLs and the eluates of the affinity purification were probed with streptavidin. Again, successful protein biotinylation was catalyzed by PsbO-APEX2, and the affinity purification of biotinylated proteins was highly efficient as strong signals were displayed in both input and the eluates, but not in the flow through(Fig. 4*B*). Note that the WT cells treated with BP and the PsbO-APEX2 cells without BP treatment were also included as the control to evaluate the background protein biotinylation (Fig. 4*B*). In the case of the WT cells treated with BP, weak protein biotinylation was detected due to the existence of the endogenous peroxidase activity (36). The almost negligible weak biotinylation signal of the control indicates that the PsbO-APEX2-catalyzed protein biotinylation is highly specific. The efficiency of affinity purification was further confirmed with the visualization of the enriched proteins stained with Coomassie blue or silver staining (supplemental Fig. S1), and much less and weaker protein bands were detected in the controls.

PsbO locates at the thylakoid lumen and PsbO-APEX2 is expected to catalyze the biotinylation of luminal proteins or the thylakoid membrane proteins with domains exposed to the lumen, but not the cytoplasmic proteins or the thylakoid membrane proteins without a domain exposed to the lumen. To examine such a spatial specificity of the labeling, the eluates of the affinity purification were probed for three proteins including PsbO, D1, and CpcB. The result showed that high spatial specificity was achieved as indicated by the detection of PsbO and D1 protein but not CpcB in the eluates for PsbO-APEX2 treated with BP (Fig. 4*C*). D1 is an integral membrane protein (IMP) with multiple transmembrane domains (TMs) and therefore have multiple inter-TM loops as well as the C terminus exposed to the thylakoid lumen (37) and is accessible for proximity labeling by PsbO-APEX2. CpcB is a subunit of phycobilisome that locates at the cytoplasmic side of the thylakoids and is not accessible for PsbO-APEX2 catalyzed labeling.

All thylakoid luminal proteins are translated outside of the lumen and transported into the lumen *via* different mechanisms (38). Although PsbO and PsbO-APEX2 as well mainly locate at the thylakoid lumen, existence of low amount of newly synthesized PsbO-APEX2 in the cytoplasm is possible. To further exclude the proteins labeled by such a particular fraction of PsbO-APEX2, a knock-in mutant with APEX2 fused at the C terminus of glucose-6-phosphate dehydrogenase, a known cytoplasmic protein encoded by the gene *zwf* (Zwf-APEX2) (11), was generated. The proteins biotinylated by Zwf-APEX2 were effectively purified by affinity purification and detected by streptavidin blotting, which show a distinct pattern from those biotinylated by the PsbO-APEX2 (Fig. 4, *B* and *D*), suggesting that a different set of proteins were labeled. Therefore, the Zwf-APEX2-labeled proteins were also included as a negative control to remove potential cytoplasmic background. Using the combination of multiple negative controls would ensure high confident identification of thylakoid lumen specific proteins within the proximity of PsbO.

### Quantitative Identification of the Proteins Proximal to the OEC

**Fig. 5:**
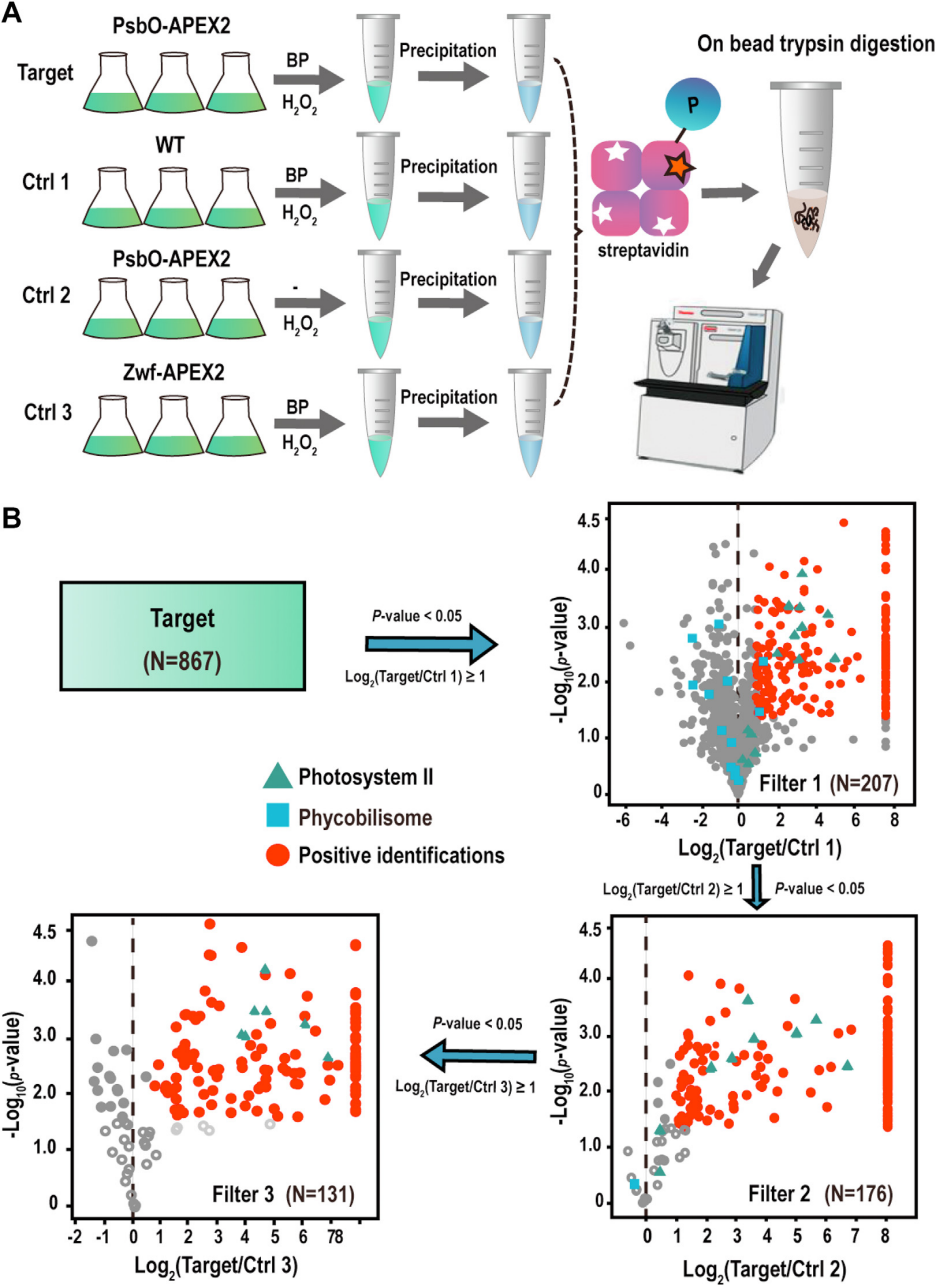
**Quantitative identification of the proteins proximal to OEC.***A*, schematic representation of the workflow for quantitative proteomic identification of the proteins proximal to the OEC. Three negative controls (Ctrls 1–3) were included. *B*, the diagramed workflow illustrates the step-by-step filtration to remove false positive identifications using the indicated criteria. The 867 proteins identified in the target experiment were sequentially filtered and only 131 proteins remained. The x-axis in each volcano plot indicates the logarithm-transformed ratio of the relative abundance of a protein between the two indicated samples. The y-axis: the logarithm-transformed *p*-value of the Student’s *t* test. The subunits of PS II were used as the positive landmarks which remained in the list after the filtering, and the subunits of phycobilisome were used as the negative landmarks which were removed in the first two filtering steps. The number of remaining proteins in each step are shown in parenthesis. OEC, oxygen evolution complex.

The strategy for the identification of proteins proximal to PsbO is illustrated in a diagram (Fig. 5*A*). The mutant cells expressing PsbO-APEX2 or Zwf-APEX2 were incubated with 2 mM BP for 10 min and followed by incubation with 2 mM H_2_O_2_ for 1 min. The WT treated in the same way was included as the control, and the mutant expressing PsbO-APEX2 incubated with H_2_O_2_ but without BP was included as an additional control as described above. Three biological replicates of each treatment were included for label-free quantitative proteomic comparison (39). The biotinylated proteins in each treatment were purified by streptavidin affinity purification as described above (Figs. 2 and 4) and then on-bead digested by trypsin (27). The tryptic peptides were subsequently analyzed with LC-MS (Fig. 5*A*).

The raw MS files were searched against the *Synechocystis* proteome database using MaxQuant (version 1.6.0.16). In total, 1202 proteins were identified with a 1% false discovery rate (supplemental Table S2). Only proteins identified in at least two replicates of the target experiment and with MS/MS count greater than one were included for further analysis. Filtered with this criterion 867 proteins were identified as the biotinylated proteins labeled by PsbO-APEX2 (Fig. 5*B*). The reproducibility of the quantitation was evaluated by the pairwise comparison of the MS intensities among the triplicate samples. In all comparisons, high correlation coefficients were obtained with a minimum R^2^ equal to 0.94, indicative of high reproducibility of the quantitation (supplemental Fig. S2).

To narrow down the number of positive identifications, the 867 proteins were sequentially compared with the proteins identified in the three negative controls to remove background identifications step by step. Student’s *t* test *p* < 0.05 and a fold-change of two were used as the criteria to filter for positive identifications in each comparison (Fig. 5*B*). In each filtering step, a significant portion of proteins were removed as illustrated in the 2D-scatter plots. Such a multistep sequential filtering with the stringent criteria resulted in 131 remaining proteins (Fig. 5*B* and supplemental Table S3). All the three subunits of the OEC (PsbO, PsbU, and PsbV) were included in the list which can be used as the positive controls. All the subunits of the phycobilisome, which were used as the negative controls, were excluded from the list. As such, the 131 proteins were determined as the proteins proximal to PsbO-APEX2 with high confidence.

### Enriched Functions in the Proteins Proximal to the OEC

**Fig. 6:**
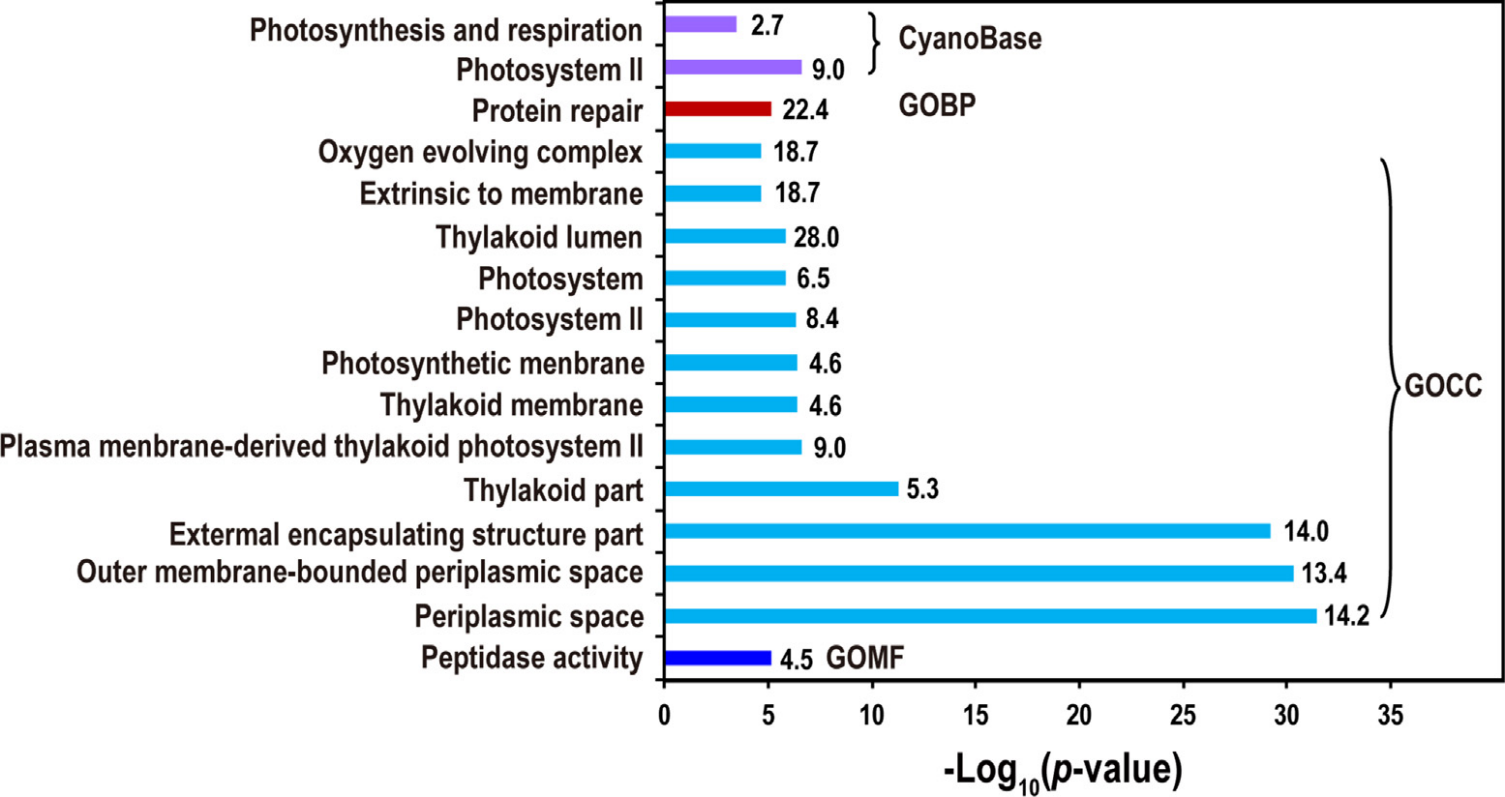
**Functions enriched in the identified proteins proximal to the OEC.** The enrichment assay was performed using Fisher’s exact test, and an enrichment factor >2 and a *p*-value < 0.05 were used together as the cutoffs. Bars show the significance of enrichment (*p*-value), and the enrichment factor was also shown to the right of each bar. CyanoBase: the functional categories in CyanoBase. GOBP, GOCC, and GOMF are the three types of gene ontologies. OEC, oxygen evolution complex.

The enriched functions among the 131 proteins were examined using Fisher’s exact test against Gene ontology terms and CyanoBase functional categories (40) (Fig. 6). For the CyanoBase functions, only two categories are enriched, namely, photosynthesis and respiration and PS II. For Gene ontology terms, one term in biological process (GOBP) and one term in molecular function (GOMF) are enriched, which are protein repair and peptidase activities, respectively (Fig. 6). The data suggest that proteases are highly needed in the proximity to the OEC, either for protein processing such as cleavage of the C-terminal tail of the precursor of the PS II reaction center D1 protein (pD1) by the protease CtpA (41), or for protein quality control such as degradation of photodamaged D1 by FtsH2/FtsH3 (42). In contrast, a number of terms in cellular component (GOCC) are enriched. The majority of the GOCC terms are related with thylakoid, thylakoid lumen, and particularly PS II but not PSI (Fig. 6). This result strongly suggests that the specificity of the current identification of proteins proximal to PS II OEC is high. Remarkably, periplasmic space is one of the most significantly enriched GOCC terms (Fig. 6), indicating that many proteins proximal to OEC are also residents in periplasm.

### Integral Membrane Proteins Proximal to the OEC

**Fig. 7:**
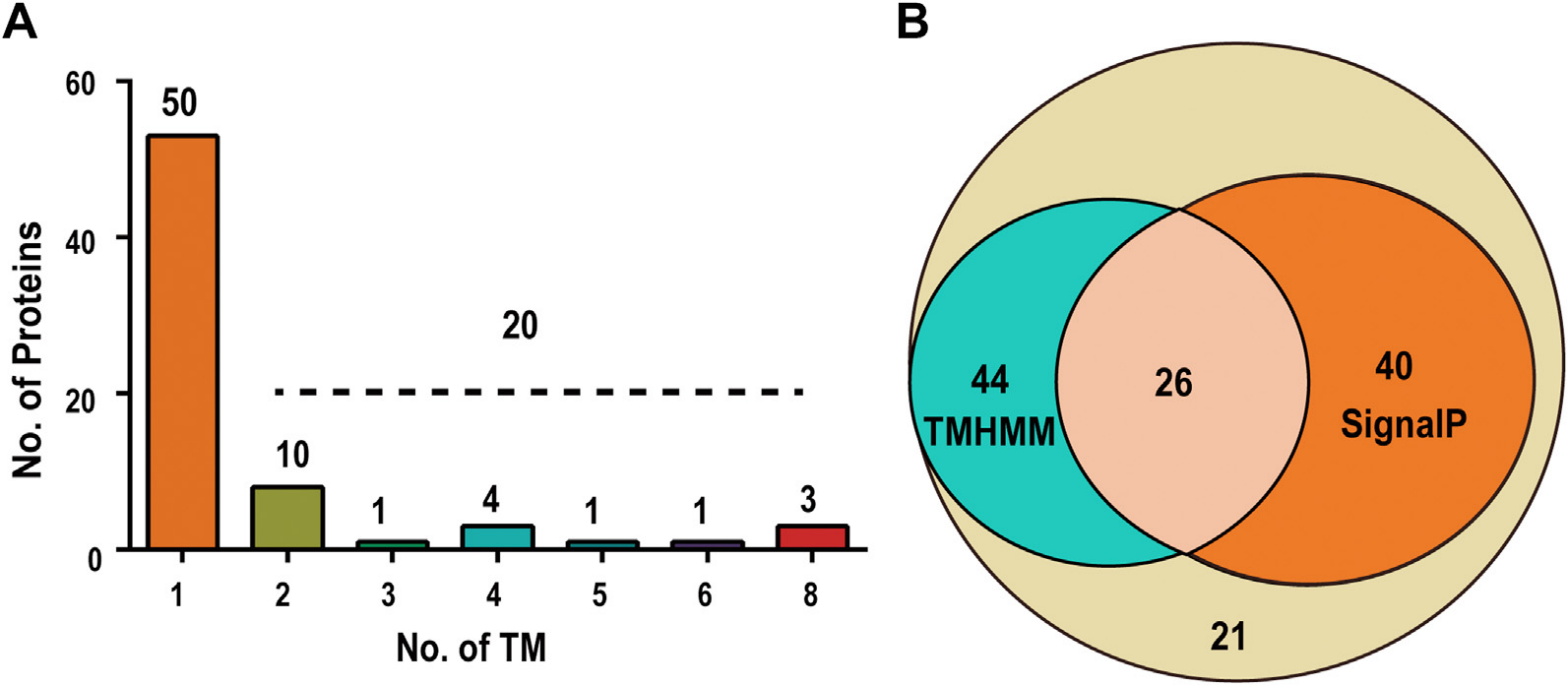
**Prediction of integral membrane proteins and luminal proteins that are proximal to the OEC.***A*, the bar graph shows the distribution of the number of proteins containing the indicated numbers of TM. The prediction was preformed using the software TMHMM 1.0 (96). *B*, the Venn diagram shows the numbers of TM-containing proteins, signal peptide-containing proteins, and proteins contain neither TM nor signal peptide. Signal peptide prediction was performed using the software SignalP 5.0 (97). OEC, oxygen evolution complex; TM, transmembrane domain.

Since the identified proteins contain both bona fide luminal proteins and membrane proteins with domains exposed to the lumen, it is important to separate these two types of proteins. Topology analysis using the program TMHMM predicts that 70 proteins contain at least 1 TM, including 20 proteins with two or more TMs (Fig. 7*A*). Signal peptide prediction for proteins with less than two TMs reveals that 66 proteins contain signal peptide at the N terminal, including 26 proteins that were also predicted as 1-TM containing (Fig. 7*B*). For 26 overlapping proteins that are predicted as both signal peptide-containing and 1-TM-containing proteins, the predicted regions for signal peptide and TM are almost completely overlapping for each protein. Since predictions of both signal peptide and TM are essentially based on the existence of a hydrophobic core, despite there may be some differences in details such as the special amino acids needed at the N terminus of signal peptide-containing proteins (38, 43), it is not easy to determine whether they are IMPs or bona fide luminal proteins solely based on computer prediction. Therefore, we also include literature evidence for the determination of true IMPs among those containing 1 TM or a signal peptide at N terminal (9, 11). Together, 38 proteins were determined as IMPs, and 93 proteins were determined as luminal proteins and collectively referred to as luminal proteins. These also include 21 proteins that contain neither predicted TM nor signal peptide. The latter may be transported to the lumen *via* an unknown pathway (Fig. 7*B* and supplemental Table S3) (38).

It is conceivable that IMPs are less mobile than soluble proteins as their horizontal movements on the membrane are restrained by the surrounding lipids. Therefore, the identified IMPs better represent the *in vivo* residents in the proximity to the OEC complex, within a small APEX-labeling radius (<20 nm) (16). The OEC neighborhood IMPs can be categorized into four groups (supplemental Table S3). First, the PS II subunits. These include D1, D2, and PsbL. Second, proteins involved in PS II assembly, maturation, and damage-degradation-repair cycle. These include HliD, YidC, Slr0575, FtsH2, FtsH3, and Psb32. HliD is a small one-TM containing membrane protein belonging to the high-light–induced protein family and was found to be associated with RCII complex and is involved in early stage assembly of PS II (44, 45). Moreover, HliD forms a complex together with chlorophyll synthase and the general insertase YidC and potentially involved in delivering chlorophyll to nascent apoproteins of PS II pigment–protein complex (46). YidC (Slr1471) is the *Synechocystis* Albino3/Oax1 homolog and is essential for membrane integration of pD1. YidC directly interacts with D1 (47) and is essential for thylakoid biogenesis (48). The hypothetical protein Slr0575 is also involved in PS II assembly. Slr0575 locates primarily on the thylakoid membrane (11) and was identified as a subunit of the PS II assembly intermediate RCII (49). FtsH2 and FtsH3 are two proteases forming hetero-oligomeric complex that is essential for D1 degradation (42, 50). FtsH2, FtsH3, and FtsH4 locate mainly on the thylakoid membrane, whereas FtsH1 locates mainly on the plasma membrane (11). FtsH4 was also identified as a OEC neighborhood protein, though its function and substrates are not well known (51). In contrast, FtsH1 was not identified. Psb32 (Sll1390) is associated with PS II and involved in protection of PS II from photodamage and acceleration of its repair (52).

Third, proteins functionally connected with PS II (supplemental Table S3). These include PetA, PetC1, CcsA, CcsB, Slr0404, Sll0814, and Hik33. PetA, PetC1, CcsA, and CcsB are related with cytochrome *b*_*6*_/*f* (Cyt *b*_*6*_/*f*) complex, one of the main protein complexes of the photosynthetic electron transport locating at the downstream of PS II and the plastoquinone pool and the upstream of PS I. The apocytochrome f (PetA) and the major Rieske protein PetC1 are two components of the Cyt *b*_*6*_/*f* complex (53). PetC1 is predicted to contain a signal peptide and was experimentally determined as a twin-arginine translocation (Tat) substrate (54, 55). In higher plants, the signal peptide protein is not cleaved off after translocation and remained as a TM (56). The proteins CcsA and CcsB are important for cytochrome f biogenesis (57, 58). These data strongly suggest that the Cyt *b*_*6*_/*f* complex is closely connected with PS II not only in function but also in space. The hypothetical protein Slr0404 is the *Synechocystis* homolog of *Arabidopsis* fluctuating-light acclimation protein1 and is involved in regulation of pH homeostasis and proper induction of NPQ in chloroplasts. Inactivation of *slr0404* impaired the growth of *Synechocystis* in light-dark cycles (59). Sll0814 is also a hypothetical protein, its homolog in *Synechococcus* sp. PCC 7002 is involved in synthesis of Myxoxanthophyll, a carotenoid glycosides acting as natural surfactants, which can stabilize membranes and regulate the permeability of membranes to oxygen (60) and presumably facilitates localized release of photosynthetically produced O_2_ from the lumen. The two-component sensory histidine kinase Hik33 is a major sensor for various abiotic stresses (61). Hik33 locates mainly on the thylakoid membrane and we previously proposed that the TM-residing Hik33 is activated not directly by the extracellular signals but indirectly by the intracellular signals, such as the redox change of the PQ pool (11). Residing in the neighborhood of PS II, and of the PQ as well, may allow Hik33 to keenly sense the redox change of the PQ pool, a consequence of the electron transport from PS II.

Fourth, proteins without known functions related with PS II (supplemental Table S3). The majority of these proteins are hypothetical proteins, and their membrane localization is supported by predicted TM-containing as well reported experimental evidence (11). The functions of these hypothetical proteins are largely unknown, and it is reasonable to infer that they might be functionally related with PS II because of their short distance. Remarkably, PsaL is the only PS I subunit that was identified in the proximity to PS II. PsaL is not an integral component of PS I monomer but is essential for formation of PS I trimer (62). Freely existing PsaL but not as a subunit of PS I trimer has the chance to locate proximal to PS II. The fact no other PS I subunit was identified as the PS II neighborhood protein strongly suggests that PS II and PS I are spatially separated, with a distance longer than the radius limited by APEX2. Indeed, in chloroplast of higher plants, PS I locates mainly on the stroma lamellae, whereas PS II locate mainly on the inner stacks of grana (63). In addition, in the *Synechococcus* sp. PCC 7942, the heterogeneous distribution of the four photosynthetic complexes is determined by live-cell fluorescence imaging (64). Moreover, in *Synechocystis*, using atomic force microscopy, large areas of featureless membranes were observed around the PS I that separate PS I from the PS II and the Cyt *b*_6_/*f* complexes (65).

### Topology Prediction for IMPs Based On the Distribution of Potential APEX2 Targeting Sites

**Fig. 8:**
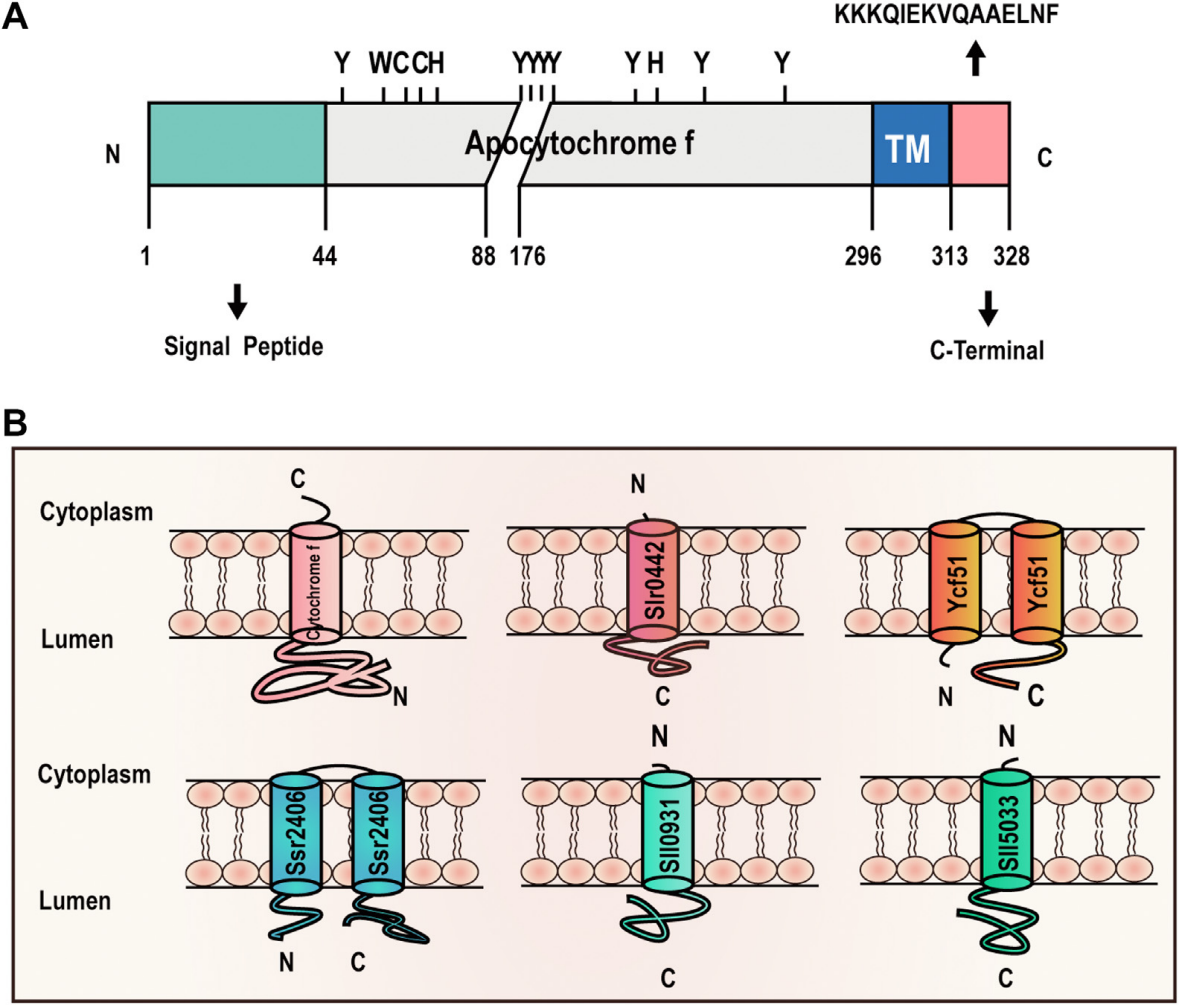
**Transmembrane topology prediction for IMPs.***A*, schematic representation of apocytochrome f structure. The N-terminal signal peptide will be cleaved when the protein become mature. The large region to the N terminal of the TM contains a plethora of potential biotinylation sites, whereas the small region to the C terminal of the TM does not contain such a site. *B*, predicted transmembrane topology for six indicated IMPs. IMPs, integral membrane proteins; TM, transmembrane domain.

The phenoxyl radicals can covalently react with electron-rich amino acids such as Tyr, Trp, His, and Cys (66, 67, 68). It was suggested that tyrosine is the primary site of biotinylation labeled by APEX2, the other three residues account for less than 2% of the APEX2-labeled residues (69). It is possible to predict transmembrane topology for certain IMPs based on the exclusive existence of such biotinylation-targeting residues in the regions exposed to the lumen. For instance, the mature cytochrome *f* has a TM locating between a long N terminus and a short C terminus (70). There are multiple potential biotin-tagging sites in the N-terminal region but not in the C terminal (Fig. 8*A*). Therefore, it is inferred that the N-terminal region of the mature cytochrome *f* locates in the thylakoid lumen while the C-terminal region locates in the cytoplasm (Fig. 8*B*). Such a topology ensures that the potential biotin-tagging sites in the N-terminal region are accessible to PsbO-APEX2 catalyzed labeling. The prediction is consistent with earlier experimental evidence (71). Similarly, we successfully predicted the topology for five other IMPs identified in the present study and two of these contain two TMs (Fig. 8*B*).

### Thylakoid Luminal Proteins in the Neighborhood of the PS II OEC Complex

The 93 luminal proteins can also be categorized into three groups based on their functional relationship with PS II (supplemental Table S3). First, the peripheral subunits of PS II or its assembly factors. These include PsbO, PsbU, PsbV, Psb27, PsbP2, and Ycf48. PsbO, PsbU, and PsbV are components of the OEC complex. Psb27 is a luminal extrinsic subunit of PS II that facilitates manganese cluster assembly (72). Psb27 also binds to CP43 complex (73, 74). PsbP2 is a peripheral component of PS II but tightly associated with the thylakoid membrane (11, 75, 76). Ycf48 is a thylakoid lumen residing PS II assembly factor that is essential for PS II assembly or stability in cyanobacteria (77). The *Arabidopsis* homolog of Ycf48 is also located in the thylakoid lumen and essential for PS II assembly (78).

Second, proteins functionally related with PS II (supplemental Table S3). These include PratA, CtpA, HhoA, PetE, MncA, and CucA. PratA is a well-documented protein important for PS II biogenesis and interacts with D1 protein (79, 80, 81, 82). It is of note that PratA was also reported as a periplasmic protein (11, 12). CtpA is a C-terminal processing peptidases for the D1 protein and functions in the thylakoid lumen (41). In most oxygenic photosynthetic organisms, D1 is synthesized as a precursor protein (pD1) with a short C-terminal extension. After integration into the thylakoid membrane, the pD1 C-terminal extension is removed by CtpA to generate mature D1 (mD1), which provides a docking site for the manganese cluster of the OEC (83). The other two carboxyl-terminal peptidases (CtpB and CtpC) were also identified in the lumen. The physiological function of the two peptidase is yet unknown, and inactivation of *ctpC* gene seems to be lethal for the cyanobacterium (84). HhoA (Sll1679) is a Deg family peptidase and is responsible for PsbO degradation in a thioredoxin-dependent manner (85). HhoA is co-transcribed with a downstream open reading frame (*sll1680*) that was also identified as a luminal protein (86). PetE (plastocyanin) is a luminal copper protein and one of the soluble electron carriers for photosynthetic and respiratory electron transport (87, 88). The heme protein PetJ (cytochrome c553) is the other soluble electron carriers in the lumen. PetJ was not identified possibly due to its low expression in the growth condition used for the present study. MncA (Sll1358) is the only known Mn^2+^-binding protein in cytoplasm and presumably involved in Mn^2+^ storage and homeostasis (79, 89). MncA is predicted to contain a TM by TMHMM. Existence of this protein in lumen indicates its role in luminal Mn^2+^ storage and homeostasis that is critical for optimal functioning of the OEC complex. Similarly, Sll1785 (CucA) is a major copper binding protein that might be important for luminal copper homeostasis and supplies copper to the other copper proteins such as PetE (89). Notably, both MncA and CucA were previously identified as periplasmic proteins (11, 12), indicating that the two proteins locate in multiple subcellular compartments.

Third, proteins without determined functional relationship with PS II (supplemental Table S3). All proteins except those in the above described two groups are assigned to this group. Notwithstanding their functional relationship with PS II remains to be determined, a few proteins have the potential to functionally interplay with PS II. One example is the existence of multiple redox proteins in the proximity to the OEC, including the thioredoxin-like proteins Slr1796, Sll1980, and a methionine sulfoxide reductase Sll1680 (MsrB). Existence of these redox proteins might be required for keen redox regulation of PS II subunits, which are highly susceptible to oxidative stresses (90, 91). Notably, Sll1980 is closely related to a thioredoxin-like protein HCF164 of *Arabidopsis* that is located in the thylakoid lumen and has disulfide reductase activity (92). Sll1680 is possibly co-expressed with HhoA under the control of the same operon (86), and co-existence of the two protein in the proximity to OEC is a strong indicator for their functional connection and is probably involved in protection against an oxidative stress and protein quality control. The other example is the existence of multiple peptidyl-prolyl cis-trans isomerases (PPI) including Sll0227, Sll0408, and Slr1761. The PPI proteins accelerate protein folding by catalyzing the cis-trans isomerization of proline (93). *Synechocystis* genome encodes only four PPI proteins (23), and three of them were identified here. Consistently, the majority of the signal peptide-containing PPI proteins were also identified in the chloroplast lumen of *Arabidopsis thaliana* (94). Existence of the PPI proteins in the thylakoid lumen is an expected observation because they are needed to accelerate protein folding, considering that many luminal proteins, including PsbO are transported across the thylakoid membrane *via* the secretory (Sec) pathway in an unfolded state (38). Probably for the same reason, Sll0227 and Slr1761 also locate in the periplasmic space (11, 12).

## Discussion

In the present study we targeted APEX2 to the *Synechocystis* thylakoid lumen through fusion with PsbO and labeled proteins in the neighborhood of the fusion protein with biotin for subsequent affinity purification and proteomic identification. In total, we identified 131 proteins proximal to PsbO-APEX2, which includes 38 integral thylakoid membrane proteins and 93 luminal proteins. It is worth noting that the identified proteins do not necessarily include either the entire neighborhood integral thylakoid membrane proteome or the entire luminal proteome. For IMPs proximal to PsbO-APEX2, only those with lumen-facing domains that contain potential sites for biotinylation were identified. For luminal proteins, labeling by PsbO-APEX2 catalyzed biotinylation could be affected by free-diffusing ability in the lumen except for those tightly associated with the OEC. Nevertheless, our results unveiled the luminal side neighborhood proteome, for the first time, for PS II in *Synechocystis*. Besides, our results showcase the feasibility to define the proteome in a subdomain of interest in this model organism. In addition to PsbO-APEX2, we also included Zwf-APEX2 in this study, though as a negative control to remove cytoplasmic background. In our practice, many APEX2-fused *Synechocystis* proteins can be expressed and efficiently catalyze proximity labeling, while some others are difficult to express. Therefore, expressing APEX2-fused proteins in *Synechocystis* is still a technical challenge. Removing the background is another technical challenge. In the present study, we included three different negative controls to remove background from different origins and successfully removed backgrounds resulting from endogenous peroxidase activity, endogenous substrates, cytoplasmic background, and even nonspecific binding to streptavidin beads. Among these, the most challenging is to remove the cytoplasmic background labeled by the residual cytoplasm-locating PsbO-APEX2, because the fusion protein is translated in the cytoplasm and might stay in the cytoplasm for a short time before translocated across the thylakoid membrane into the luminal destination. Because of this, it is conceivable that it is much more difficult to identify proteins proximal to a freely diffusing soluble protein than an integral or peripheral membrane protein that has a relatively stable cellular location.

During the progress of the current study, Dahlgren *et al*. published their work on the proximity labeling-based proteomic identification of luminal proteins in *Synechococcus* sp. PCC 7002 (21). Both studies chose an extrinsic subunit of PS II as the bait in coincidence. Among the 131 proteins identified by us, 95 have homologs in *Synechococcus*, and 51 of these were also identified by the other study. These include but not limited to PS II subunits and assembly factors, multiple proteases, and a number of proteins existing also in periplasmic space (supplemental Table S4). The overlapping proteins in the two studies presumably represent the consensus neighborhood proteins of the PS II OEC and corroborate the feasibility of defining the proteome within a particular domain of interest in cyanobacteria using APEX-based proximity labeling. Nevertheless, significant difference exists between the two studies in both experiment design and the results. First, we used three negative controls, and the other study used only one to exclude background. The two extra controls used in the present study were attempted to exclude the background resulting from endogenous peroxidase activity (the WT strain treated with H_2_O_2_ and BP) and the background from endogenous biotinylated proteins (PsbO-APEX strain treated with H_2_O_2_ only), the exclusion of cytoplasmic background was both attempted by using different fusion proteins, *i.e.*, Zwf-APEX2 in the present study and GFP-APEX2 in the other study. Although the background signals from the control of WT strain are hardly detectable by antibiotin blotting (Fig. 1*C*), the signals are obviously detectable when the biotinylated proteins were enriched by streptavidin affinity purification (Fig. 4*B*). Hence it is important to include these negative controls. Second, because of the difference in experiment design, it is expectable that the result is different. The present study identified 44 proteins with homologs in *Synechococcus* that were not identified by Dahlgren *et al.* (21), the majority of these are functionally uncharacterized proteins (supplemental Table S4). Similarly, Dahlgren *et al.* identified 55 *Synechococcus* proteins with homologs in *Synechocystis* that were excluded in our identification list (supplemental Table S4). These include five PS I subunits (PsaA, PsaB, PsaC, PsaD, and PsaF) and two ATP synthase subunits (AtpF and AtpG). PsaC and PsaD are the two extrinsic PS I subunits at the cytoplasmic side, they are not expected to be labeled by the luminal side APEX2 activity. This further underlines the importance to use more stringent controls to exclude background. Third, both studies uniquely identified a set of organism-specific proteins (supplemental Table S4). For example, we identified plastocyanin (PetE) but not cytochrome c553 (PetJ) whereas the other study identified PetJ but not PetE. Both PetE and PetJ are known soluble electron carriers in thylakoid lumen in *Synechocystis*, but in *Synechococcus*, only PetJ is expressed (21). Fourth, experimental procedures. We found that it is impossible to effectively enrich biotinylated proteins without removing the excess BP from the lysate of the cells underwent APEX2-catalyzed proximity labeling through filtering or precipitation of the samples (Fig. 2), and this is consistent with a previous report (35). In the other study, removal of excess BP was omitted (21). This is the only experimental procedure with major difference, and the differences of the other procedures are minor and compared side by side (supplemental Table S5). Last, the difference in the bait proteins. In the present study, PsbO is used as the bait, and in the other study, PsbU was used as the bait. PsbO and PsbU are both the subunits of the OEC, and the difference might be minor. Notwithstanding these differences, both studies generated a protein catalog that is important to define the protein neighborhood of PS II OEC in the respective model organism.

The multiple negative controls and the stringent filtering criteria allow us to resolve the PS II OEC neighborhood proteome with a resolution that can exclude the other major macromolecular protein complexes on the thylakoid membrane such as PS I and ATP synthase. This result suggests that PS I and ATP synthase as well are spatially separated from PS II with a distance beyond the labeling radius of APEX2 (∼20 nm) (16), which supports the notion that thylakoid membrane is not homogenous (64). Using the same approach, the neighborhood proteome of the other membrane or membrane-associated proteins can also be spatiotemporally resolved with a similar resolution. This information will be important for structural, functional, and physiological studies involved in both basic and applied research, particularly in discovering novel proteins that interact with and play a significant role in regulating the structure and function of a protein complex of interest.

A remarkable finding is that 57 luminal proteins were also previously identified from isolated periplasm by us and the others (supplemental Table S3) (11, 12), and similar finding was also reported by Dahlgren *et al.* (21). Such a large portion cannot be simply explained by contamination. Some of these proteins are associated with the periplasmic side of the plasma membrane, and it is not surprising that they are also associated with the luminal side of the thylakoid membrane because it was proposed that thylakoid membrane might be originated from the plasma membrane (12). In fact, a recent study also showed that the biogenic regions of the thylakoid membrane forms contacts with the plasma membrane (14). PratA is one of the proteins locates at this region (80, 82). The other proteins may be transported to the two different compartments by the endogenous protein translocation systems. In cyanobacteria, the Sec and the Tat are two major pathways transporting proteins to both periplasmic space and thylakoid lumen (38). *Synechocystis* has only one set of Sec and Tat transport systems that locate on both the plasma membrane and the thylakoid lumen (11, 12); therefore, it is possible a particular protein can be transported across the plasma membrane to the periplasmic space or across the thylakoid membrane to the lumen. If this is the case, an interesting question would be raised regarding the selectivity of the translocation because some proteins are translocated to a single destination while the others are translocated to both destinations. The other intriguing question is what the functional difference is when a particular protein locates at a different compartment.

In summary, we developed an effective workflow for APEX2-based proximity labeling in *Synechocystis* and successfully resolved neighborhood proteome of the PS II OEC. The information is particularly useful for the mechanistic study regarding the PS II structure and function.

## Data availability

The raw mass spectrometry proteomics data have been deposited to the ProteomeXchange Consortium *via* the PRIDE (95) partner repository with the dataset identifier PXD036113.

## Supplemental data

This article contains supplemental data.

## Conflict of interest

The authors declare no competing interests.
