# Supplementary material for: Proximity Labeling Facilitates Defining the Proteome Neighborhood of Photosystem II Oxygen Evolution Complex in a Model Cyanobacterium
Source: Mol Cell Proteomics. 2022 Nov 8;21(12):100440. doi: 10.1016/j.mcpro.2022.100440 (PMC9764255; doi:10.1016/j.mcpro.2022.100440)
Supplement: Supplemental Data [file mmc1.pdf]

Supplementary Materials for

Proximity labeling facilitates defining the proteome neighborhood of photosystem II oxygen evolution complex in a model cyanobacterium

Zhen Xiao [1,2,4], Chengcheng Huang [1,2,4], Haitao Ge [1], Yan Wang [1,2], Xiaoxiao Duan[1,2], Gaojie Wang[1,2], Limin Zheng[1,2], Jinghui Dong[1,2], Xiahe Huang[1], Yuanya Zhang[1], Hongyu An[1, 2], Wu Xu[3], and Yingchun Wang[1,2]*

[1]State Key Laboratory of Molecular Developmental Biology, Innovation Academy for Seed Design, CAS, Institute of Genetics and Developmental Biology, Chinese Academy of Sciences, No.1 West Beichen Rd., Beijing 100101, China.

[2]University of Chinese Academy of Sciences.

[3]Department of Chemistry, University of Louisiana at Lafayette, Lafayette, LA 70504, USA.

[4]Co-first author.

This PDF file includes:

Figures S1-S3

Supplemental data:

Fig. S1. Visualization of affinity purified biotinylated proteins in the indicated samples by coomassie blue staining.

Fig. S2. Evaluation of the reproducibility of the label-free quantitation.

Fig. S3. Annotated spectra for all proteins identified with a single peptide.

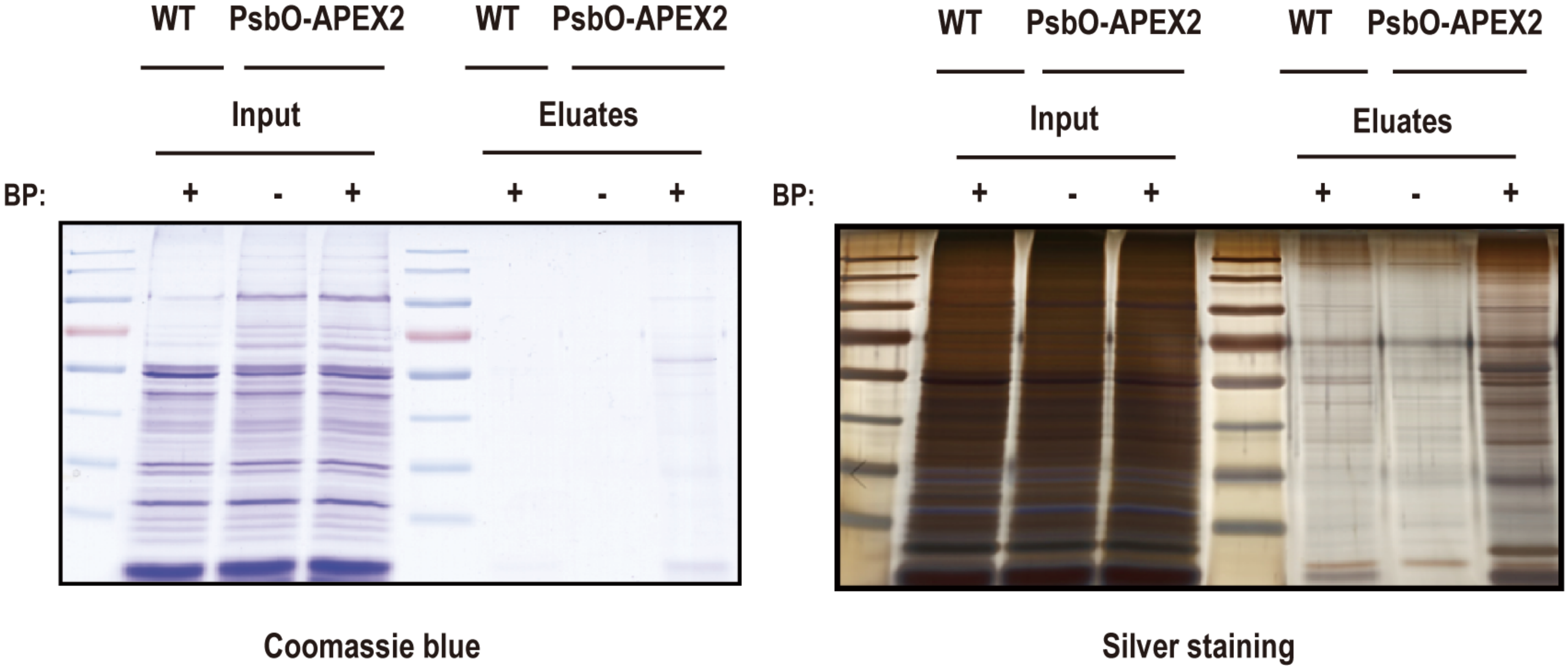

Fig. S1. Visualization of affinity purified biotinylated proteins in the indicated samples by coomassie blue staining (left panel) and silver staining (right panel).

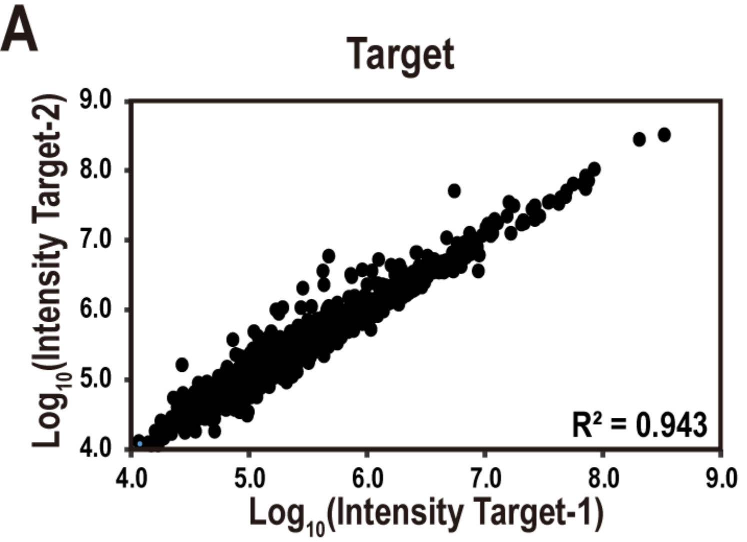

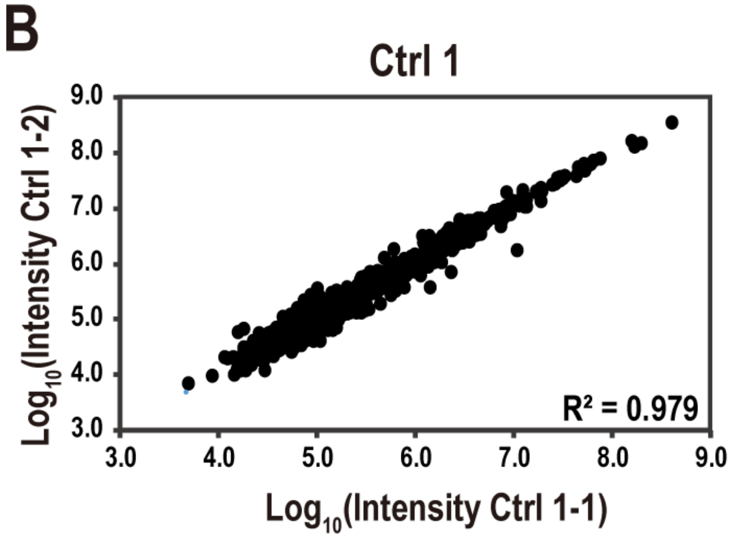

R^2 (Target)

	R1	R2	R3
R1	1		
R2	0.943	1	
R3	0.943	0.984	1

R^2 (Ctrl 1)

	R1	R2	R3
R1	1		
R2	0.979	1	
R3	0.980	0.983	1

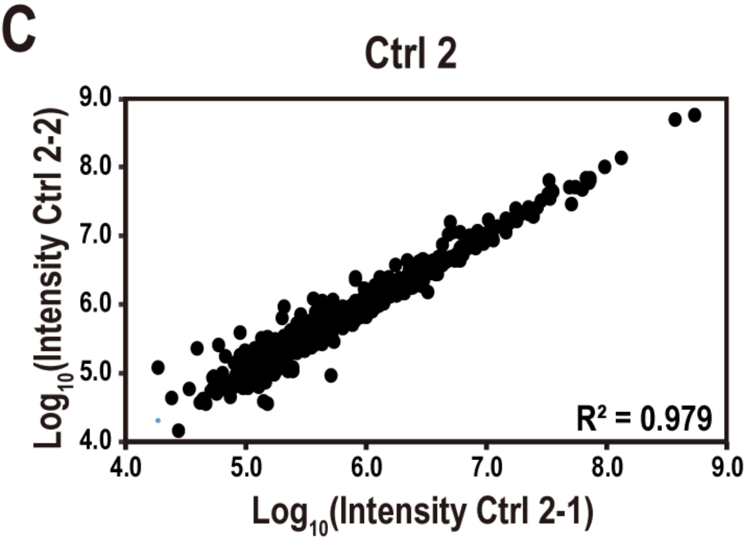

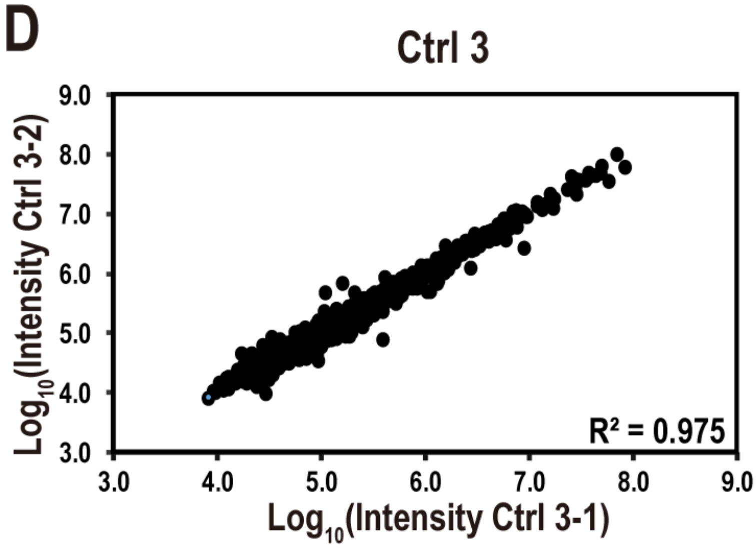

R^2 (Ctrl 2)

	R1	R2	R3
R1	1		
R2	0.979	1	
R3	0.982	0.981	1

R^2 (Ctrl 3)

	R1	R2	R3
R1	1		
R2	0.975	1	
R3	0.976	0.962	1

Fig. S2. Evaluation of the reproducibility of the label-free quantitation.
Scatter-plot show the comparison of the MS intensities for all proteins quantified for replicate 1 and replicate 2 in the samples Target and the Controls 1-3 (*A-D*, upper panels). The correlation coefficients (R^2) of the pairwise comparisons among the three replicates for each sample were shown in Tables (*A-D*, lower panels).

Raw File	Scan	Method	Score	m/z
ELITE_XZ_A-PsbO_BP_2_20210226	2306	ITMS; CID	72.43	374.53

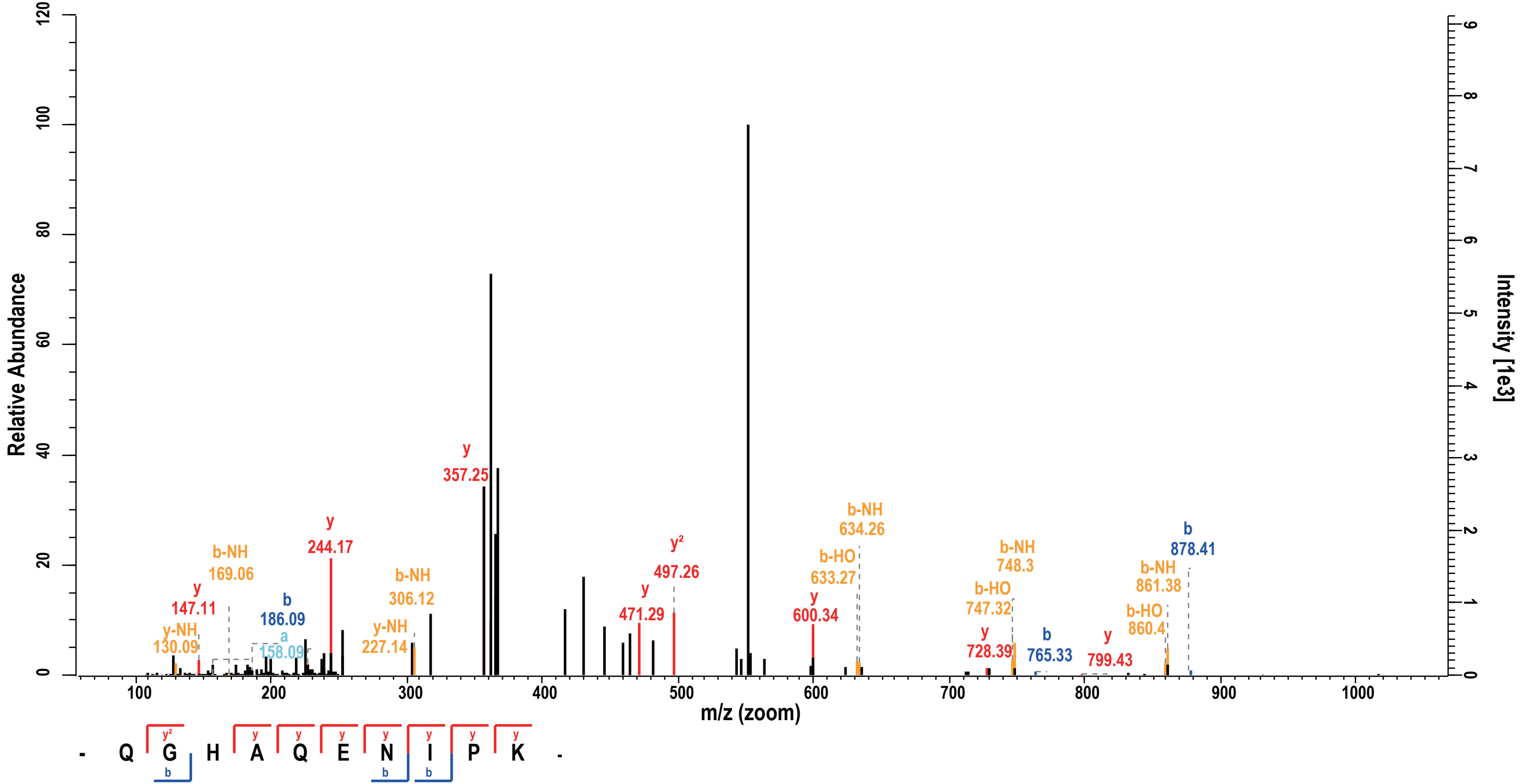

sll0383 cobalamin biosynthesis protein M

Raw File	Scan	Method	Score	m/z
ELITE_XZ_A-PsbO_BP_2_20210226	13470	ITMS; CID	75.21	436.22

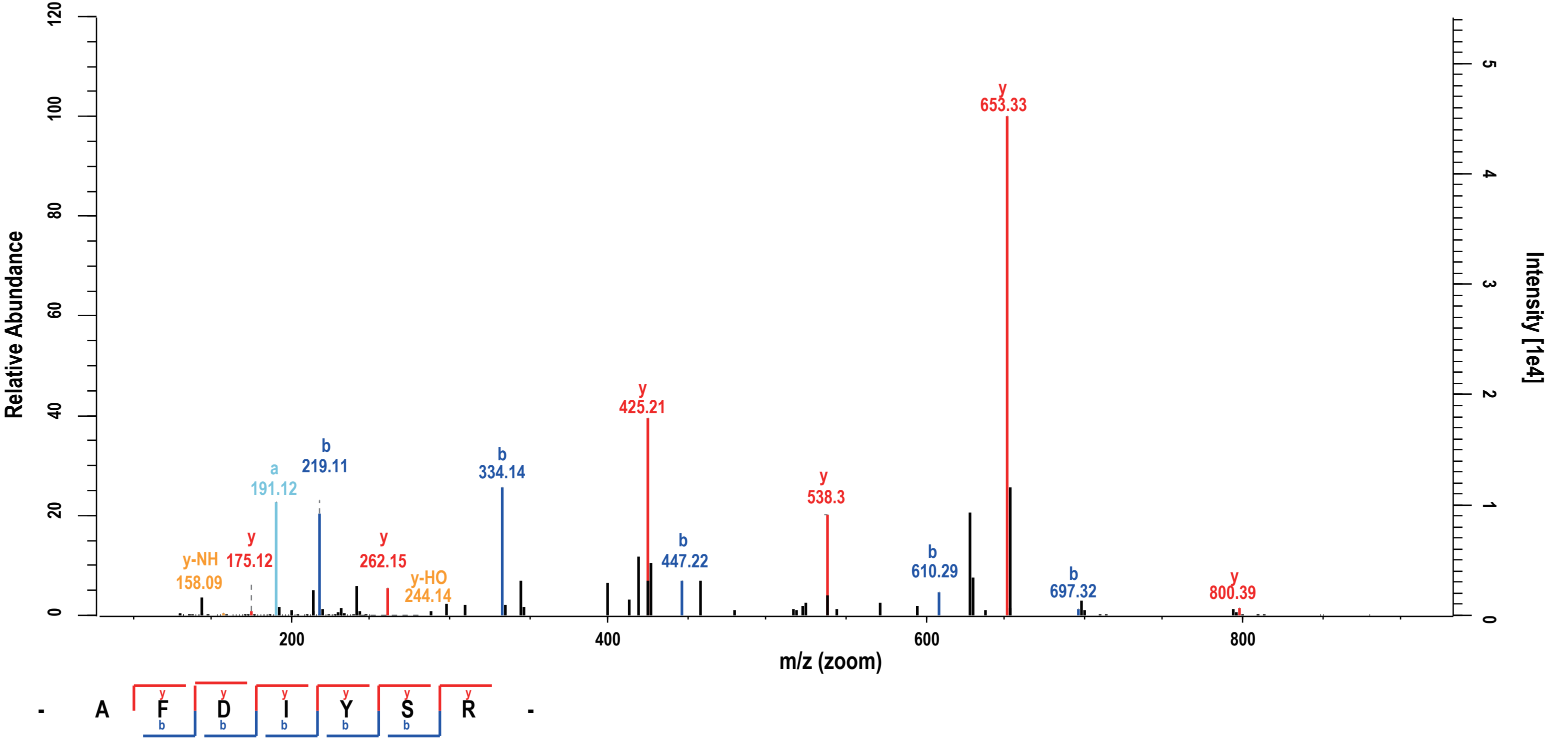

sll0534 ATP-dependent Clp protease proteolytic subunit 2

Raw File	Scan	Method	Score	m/z
ELITE_XZ_A-PsbO_BP_2_20210226	11120	ITMS; CID	158.06	826.37

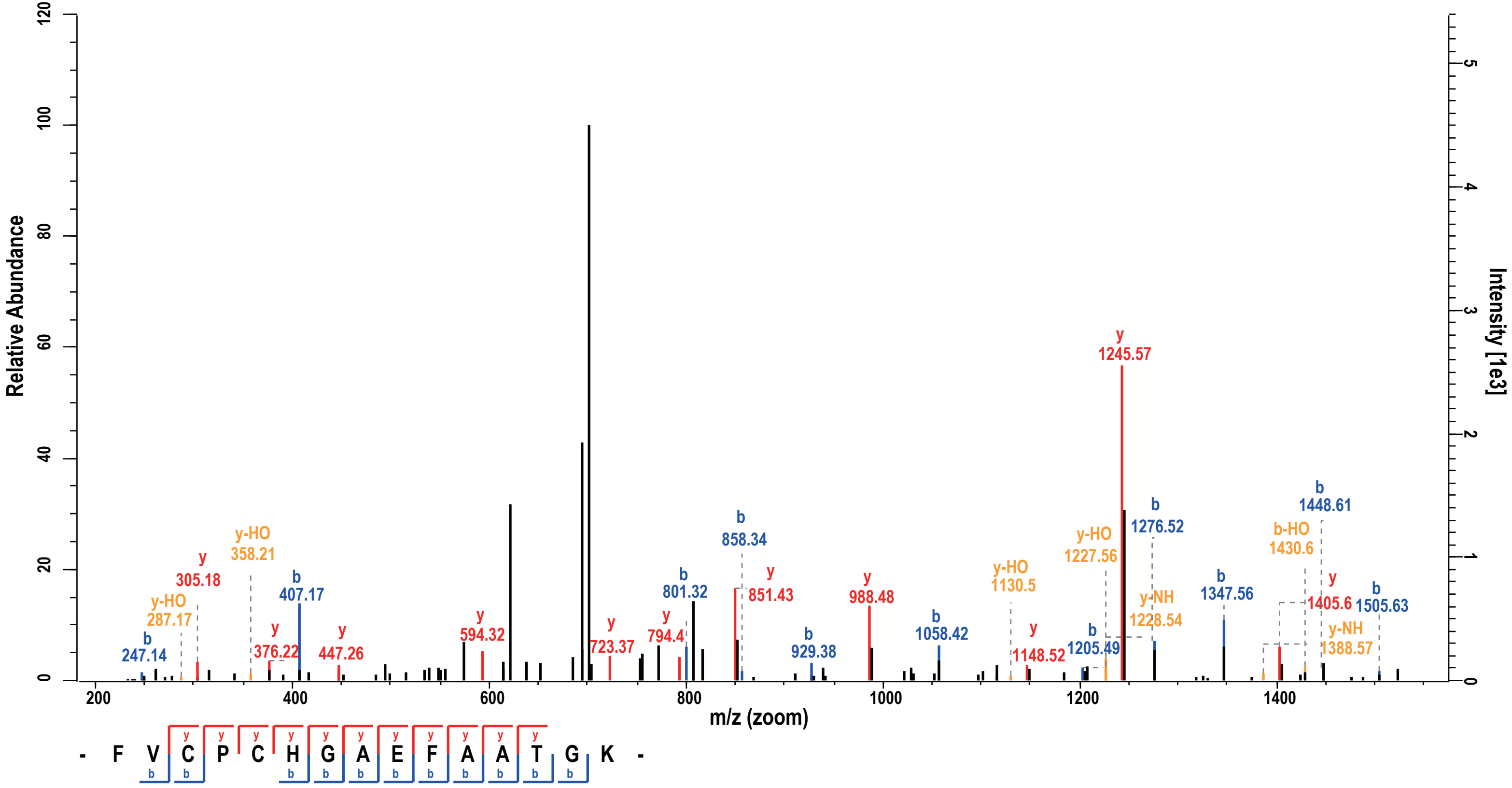

sll1182 cytochrome b6-f complex alternative iron-sulfur subunit (Rieske iron sulfur protein)

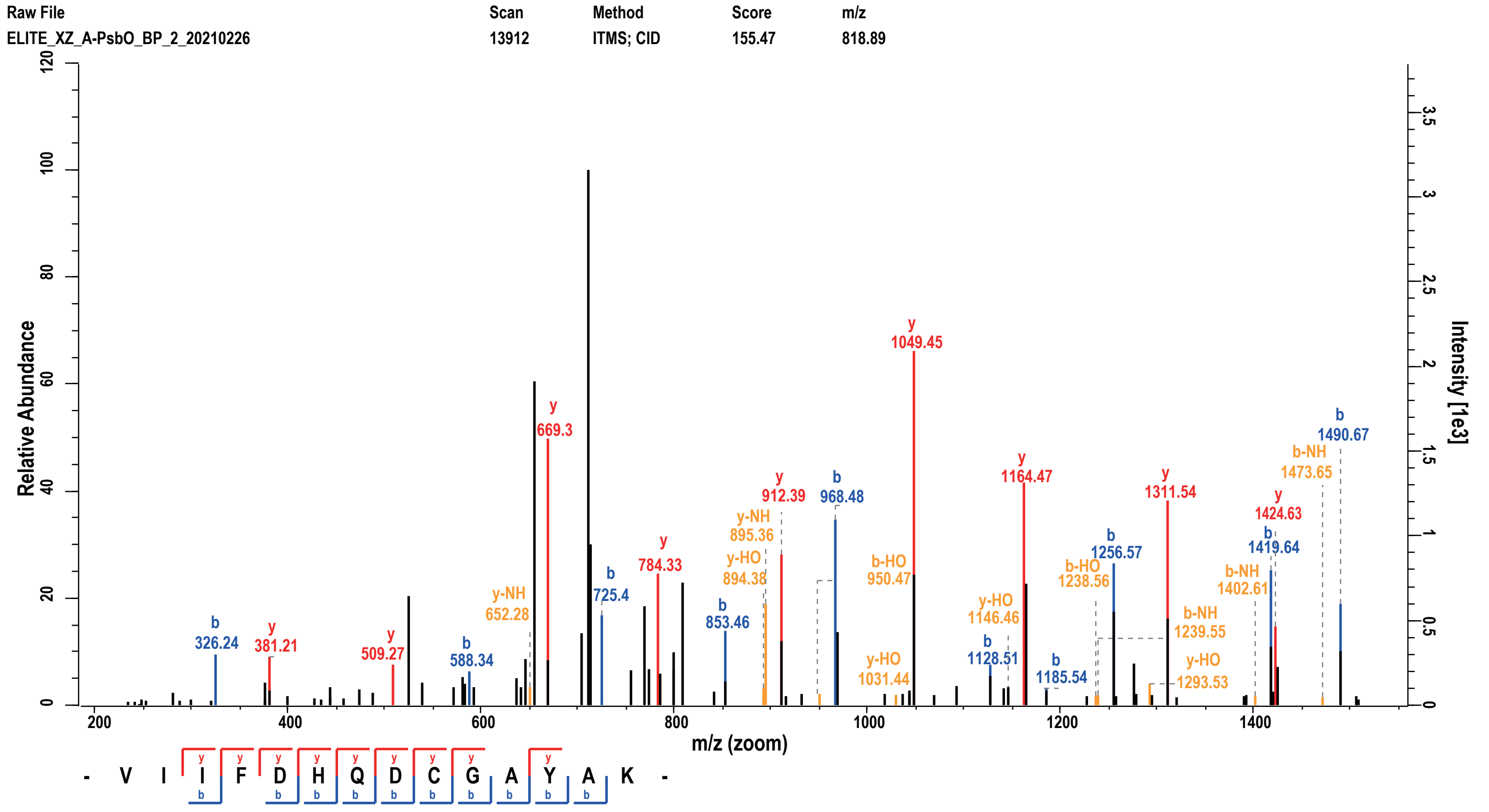

sll0732 hypothetical protein

Raw File	Scan	Method	Score	m/z
ELITE_XZ_A-PsbO_BP_2_20210226	14114	ITMS; CID	101.46	497.78

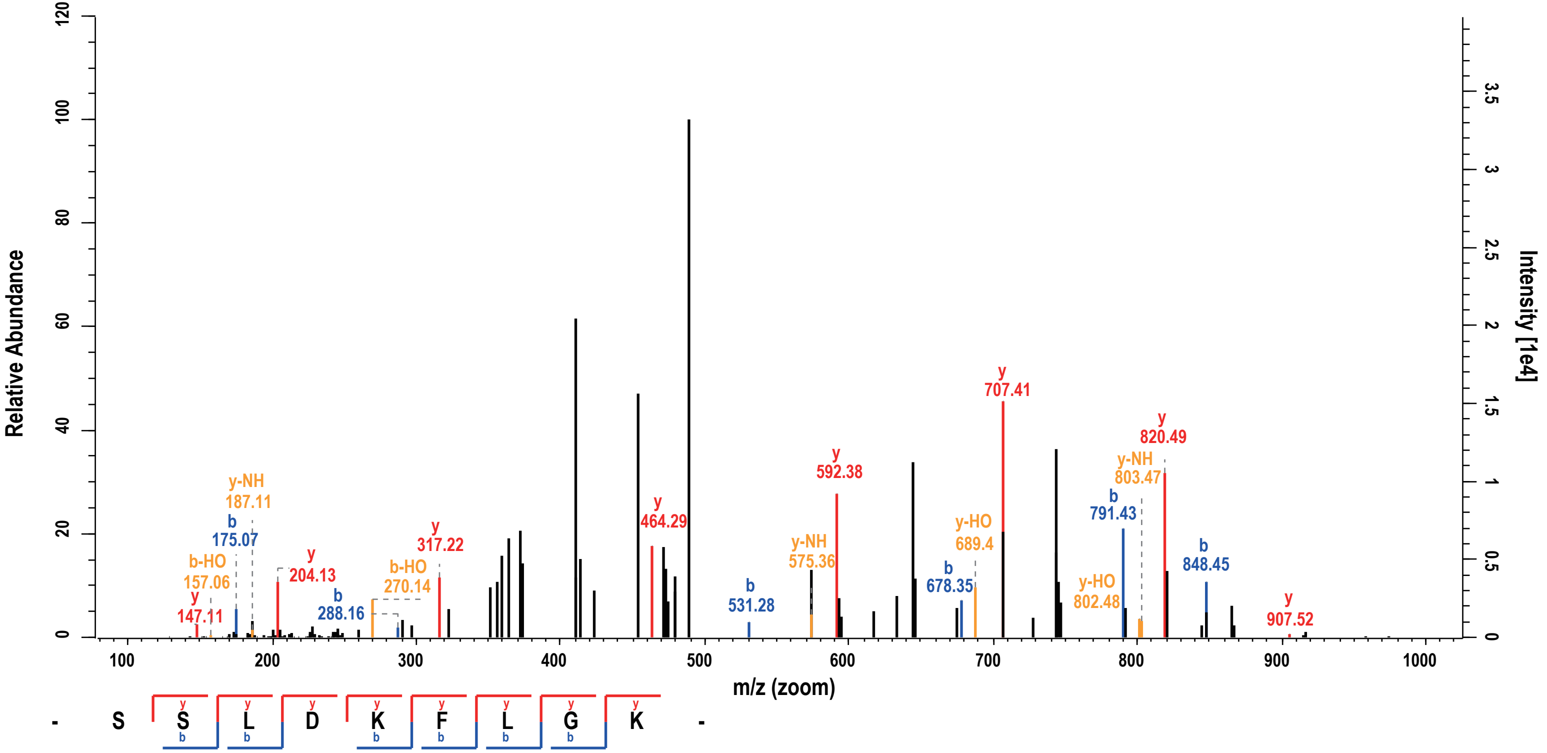

slr0680 hypothetical protein

Raw File	Scan	Method	Score	m/z
ELITE_XZ_A-PsbO_BP_2_20210226	20303	ITMS; CID	159.58	839.95

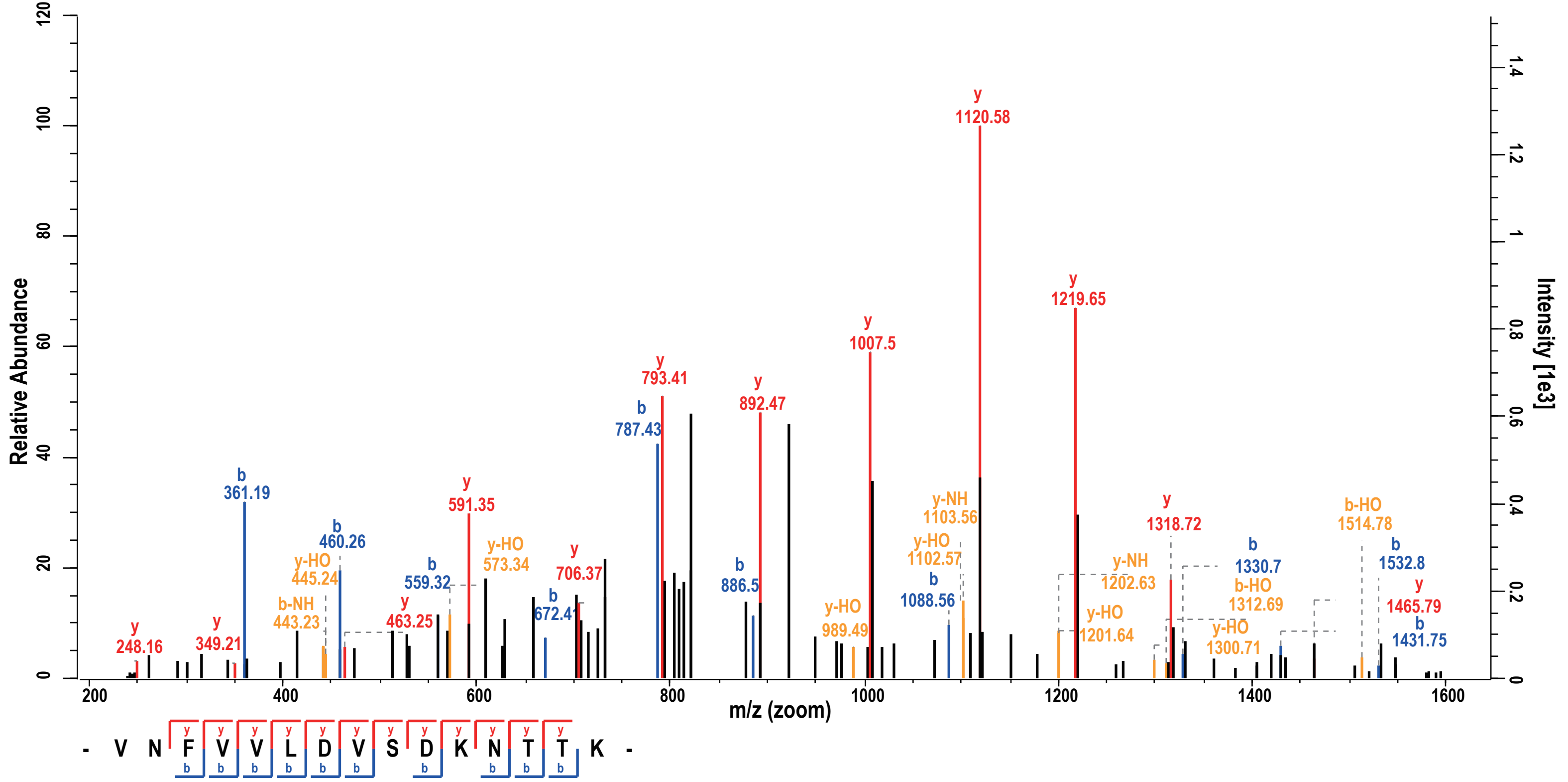

sll0685 hypothetical protein

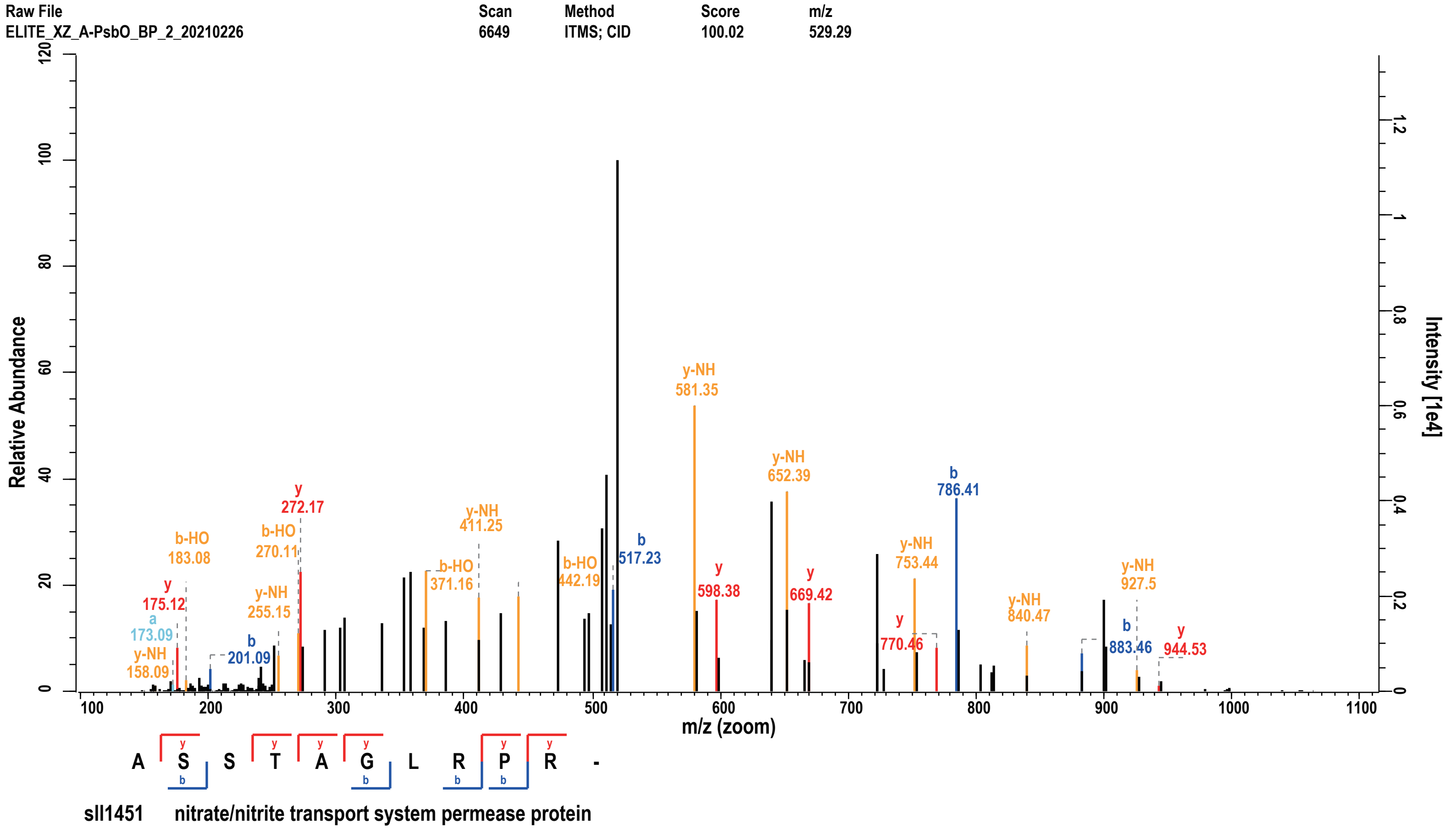

Raw File
ELITE_XZ_A-PsbO_BP_2_20210226
Scan
6649
Method
ITMS; CID
Score
100.02
m/z
529.29
Relative Abundance
Intensity [1e4]
m/z (zoom)
y-NH 158.09
a 173.09
y 175.12
b-HO 183.08
b 201.09
y-NH 255.15
b-HO 270.11
y 272.17
b-HO 371.16
y-NH 411.25
b-HO 442.19
b 517.23
y-NH 581.35
y 598.38
y-NH 652.39
y 669.42
y-NH 753.44
y 770.46
b 786.41
y-NH 840.47
b 883.46
y-NH 927.5
y 944.53
A S S T A G L R P R -
sll1451 nitrate/nitrite transport system permease protein

Raw File	Scan	Method	Score	m/z
ELITE_XZ_A-PsbO_BP_3_20210226	2392	ITMS; CID	61.78	405.88

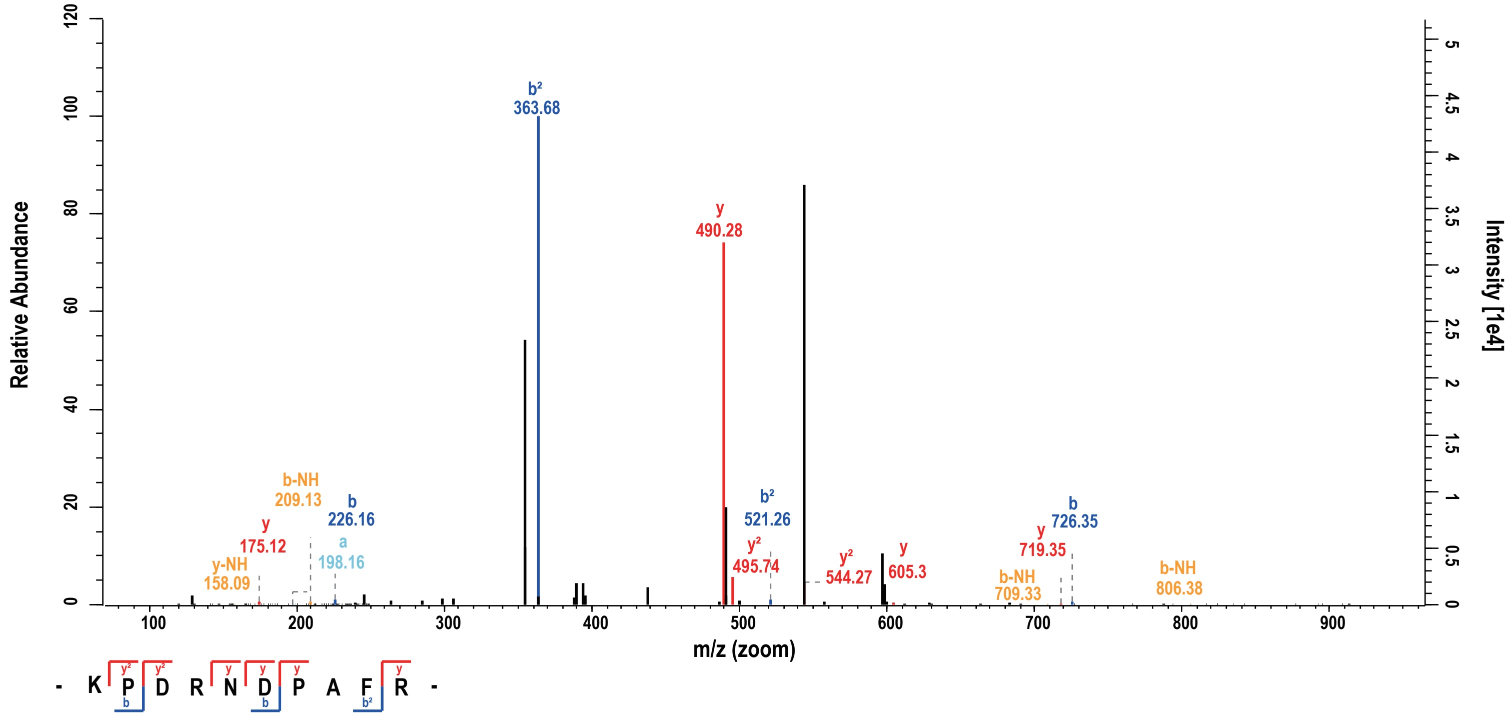

ssl3342 hypothetical protein

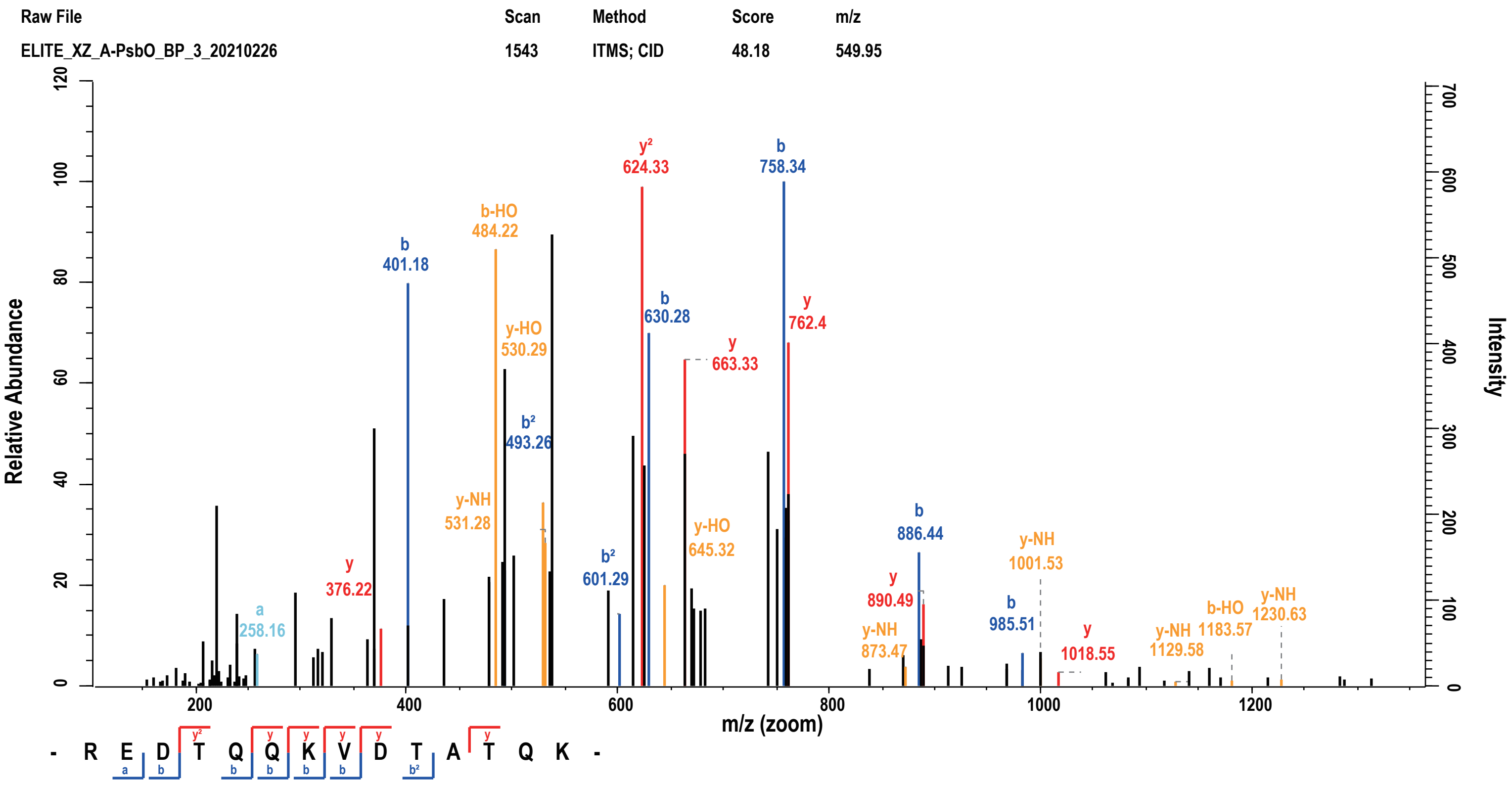

slr1596 a protein in the cytoplasmic membrane involved in light-induced proton extrusion.

Raw File	Scan	Method	Score	m/z
ELITE_XZ_A-PsbO_BP_3_20210226	22842	ITMS; CID	165.16	1325.65

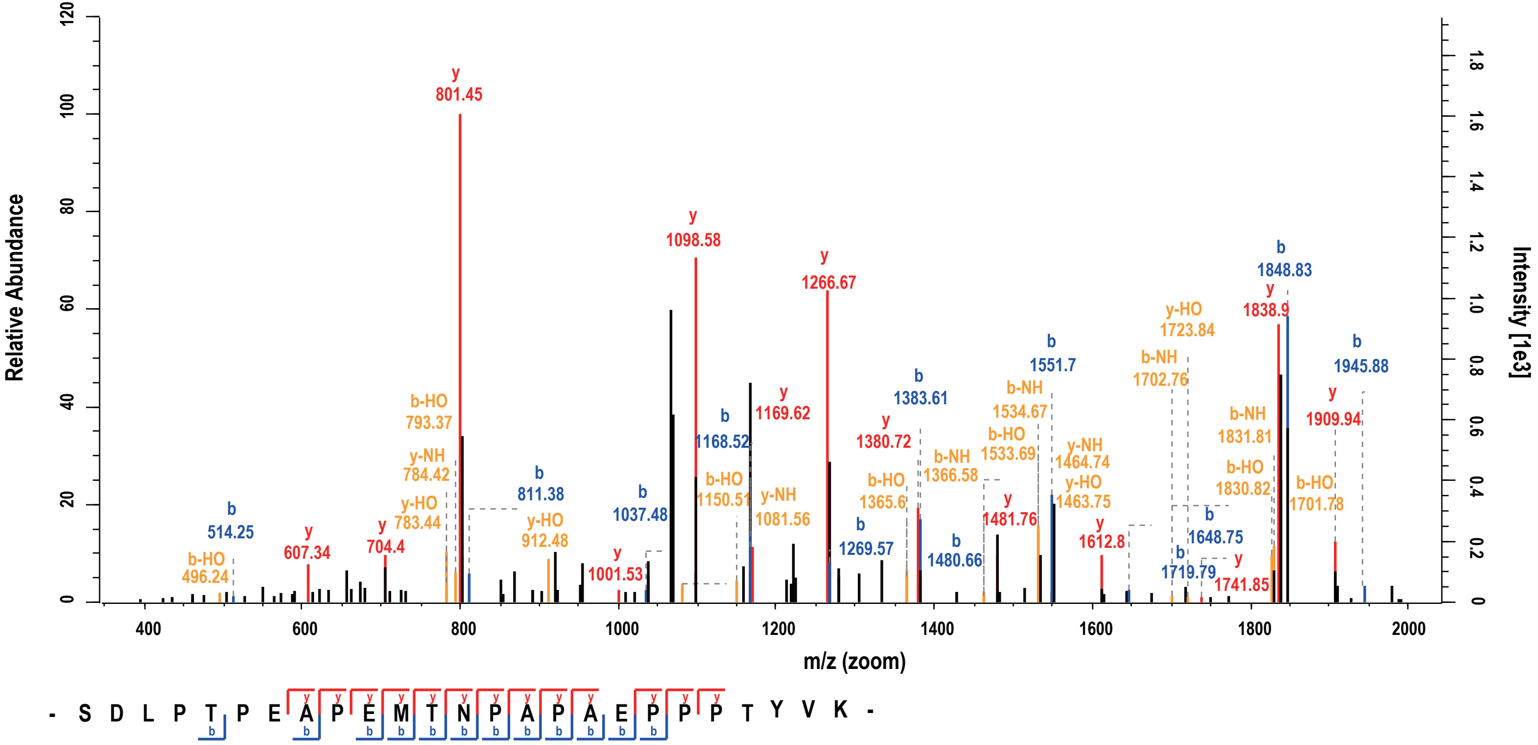

ssr0109 hypothetical protein

Raw File	Scan	Method	Score	m/z
ELITE_XZ_A-PsbO_BP_3_20210226	5237	ITMS; CID	50.87	629.33

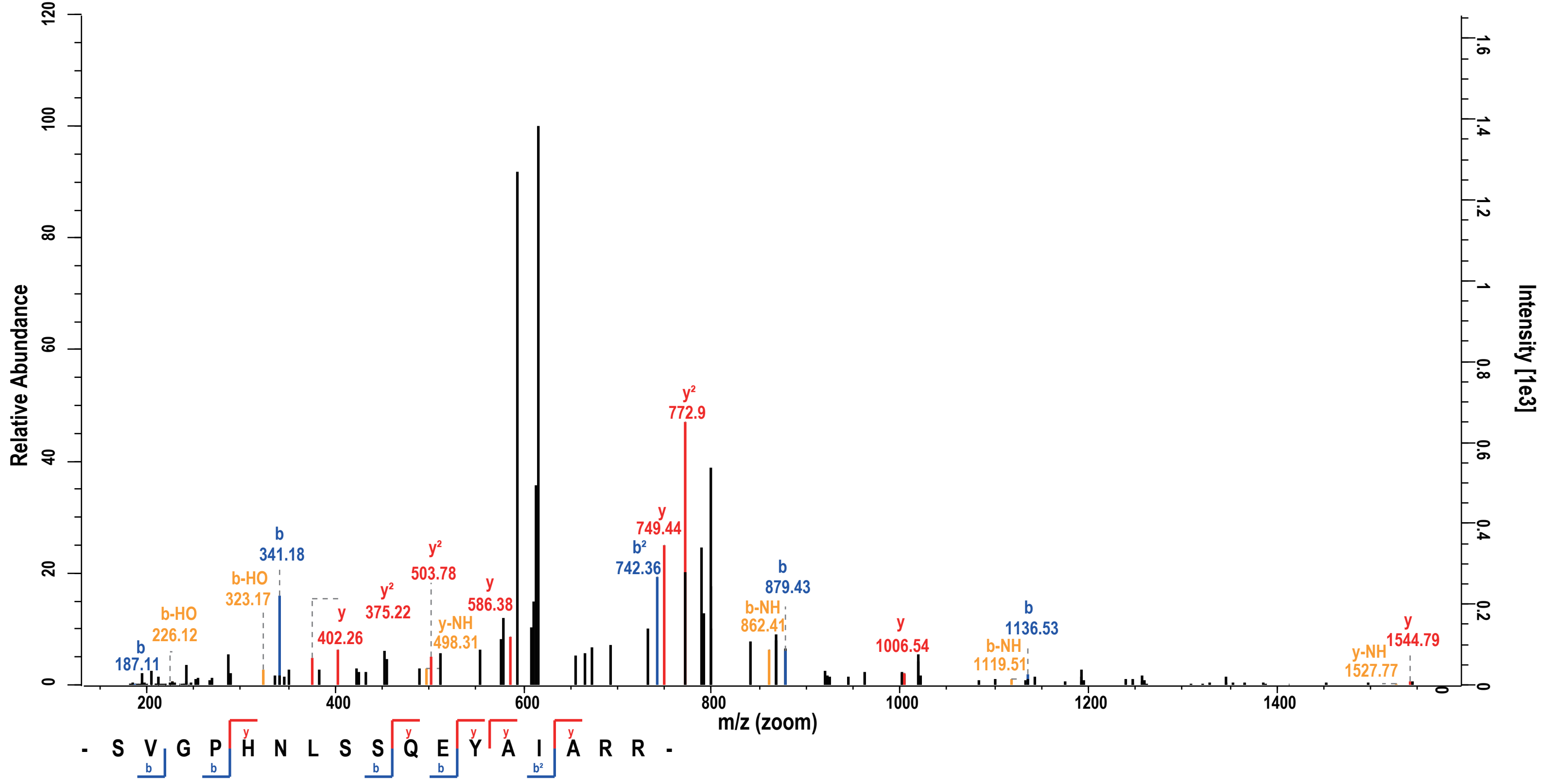

sll0916 precorrin isomerase, precorrin-8X methylmutase

Raw File	Scan	Method	Score	m/z
ELITE_XZ_A-PsbO_BP_3_20210226	24254	ITMS; CID	191.01	974.01

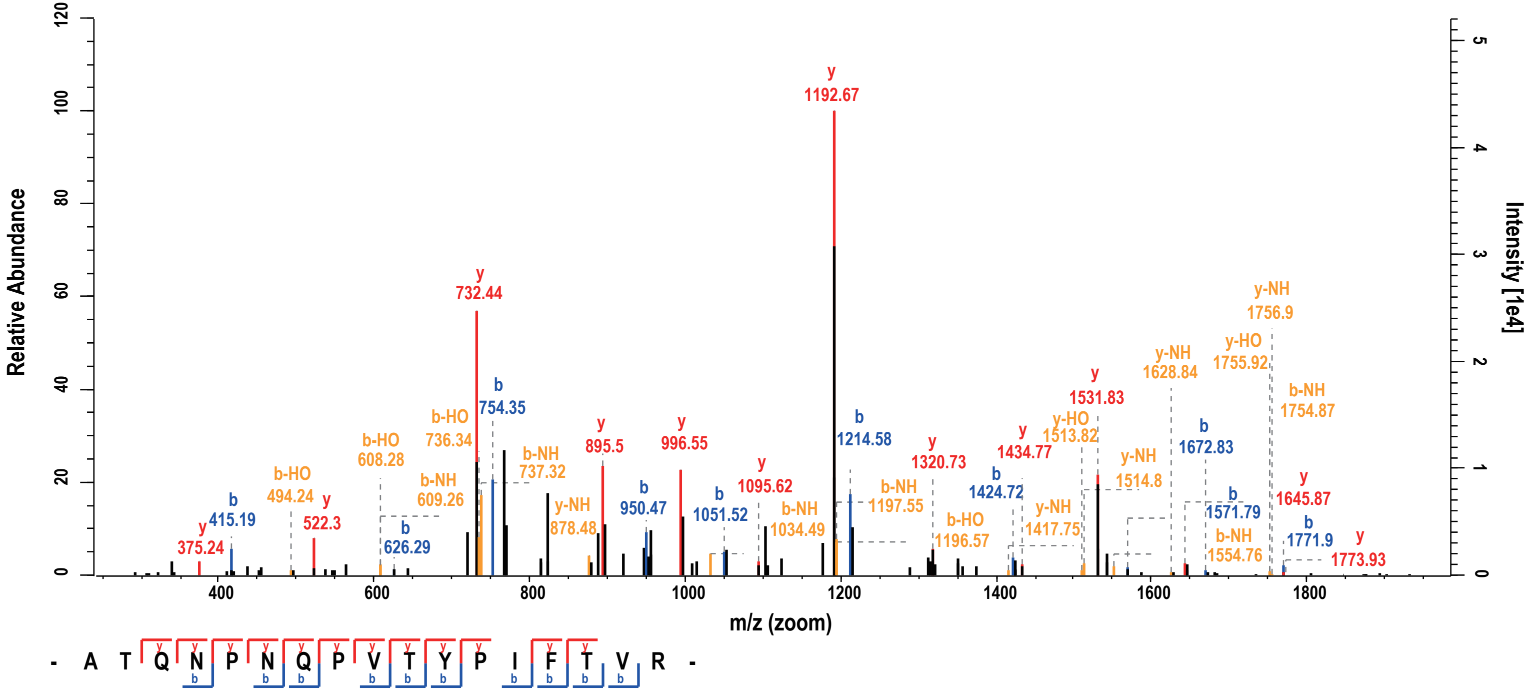

smr0006 cytochrome b559 b subunit

Fig. S3. Annotated spectra for all proteins identified with a unique single peptide.
